# Simplifying the estimation of diagnostic testing accuracy over time for high specificity tests in the absence of a gold standard

**DOI:** 10.1111/biom.13689

**Published:** 2022-05-26

**Authors:** Clara Drew, Moses Badio, Dehkontee Dennis, Lisa Hensley, Elizabeth Higgs, Michael Sneller, Mosoka Fallah, Cavan Reilly

**Affiliations:** 1 Division of Biostatistics, University of Minnesota, Minneapolis, Minnesota, USA; 2 Partnership for Research on Vaccines and Infectious Diseases in Liberia (PREVAIL), Monrovia, Liberia; 3 Department of Epidemiology and Biostatistics, University of California, San Francisco, California, USA; 4 Division of Clinical Research, National Institute of Allergy and Infectious Diseases, Bethesda, Maryland, USA; 5 Laboratory of Immunoregulation, National Institute of Allergy and Infectious Diseases, Bethesda, Maryland, USA; 6 National Public Health Institute of Liberia, Monrovia, Liberia

**Keywords:** diagnostic testing, Ebola virus disease, latent class model, multiple testing, nongold-standard test, nonparametric model

## Abstract

Many different methods for evaluating diagnostic test results in the absence of a gold standard have been proposed. In this paper, we discuss how one common method, a maximum likelihood estimate for a latent class model found via the Expectation-Maximization (EM) algorithm can be applied to longitudinal data where test sensitivity changes over time. We also propose two simplified and nonparametric methods which use data-based indicator variables for disease status and compare their accuracy to the maximum likelihood estimation (MLE) results. We find that with high specificity tests, the performance of simpler approximations may be just as high as the MLE.

## 1 | INTRODUCTION

Evaluating results of diagnostic tests in the absence of a gold standard is a critical problem in patient care and is correspondingly the subject of a large body of research. In a recent review of methodology on diagnostic accuracy where a gold standard is missing or unavailable, Chikere et al. (2019) categorize methods into four groups: (1) Methods where gold-standard results are missing, but a gold-standard test does exist; (2) Correction methods using imperfect reference tests with known diagnostic accuracy; (3) Methods using multiple results from the same or various imperfect tests with unknown diagnostic accuracy; and (4) Less robust methods such as agreement and test positivity rates. For this paper, we are interested in methods that fall into group 3. These methods are likely to be needed when evaluating diagnostic tests for emerging infectious disease viruses such as Ebola virus or more recently, SARS-CoV-2. For these viruses, true patient status is unknown. While it cannot be determined directly, some methods have been proposed approximating it using discrepancy analysis (administering a resolver test when discordant results occur) (Hawkins et al., 2001; van Dyck et al., 2004; Juhl et al., 2013) or using a composite reference standard combining multiple other test results (Alonzo and Pepe, 1999). Rather than approximating disease status with imperfect tests, latent class models (LCMs) can also be used. These models do not suffer from the same biases as discrepancy analysis (Chikere et al., 2019) or require as many assumptions about testing accuracy as a composite reference standard. Most importantly, they do not require more than one type of test to be administered to a participant. LCMs can be fit using frequentist (Hui and Walter, 1980; Qu et al., 1996; Albert et al., 2001; Chu et al., 2009a, 2009b; Xu and Craig, 2009) or Bayesian (Black and Craig, 2002; Chu et al., 2009a; Ling et al., 2014) methods. To maximize the likelihood or posterior of an LCM, the Expectation-Maximization (EM) algorithm is often employed, maximizing over both the unknown patient status and the model parameters.

To measure viral persistence of a disease, multiple applications of the same nongold-standard tests can be administered over time to a population known to be previously infected. In a study of adult, male, Ebola virus disease (EVD) survivors that serve as motivation for the work described here, participants were asked to supply samples to determine the presence of Ebola RNA in semen. This is an important public health issue as sexual transmission of Ebola virus has been documented. Survivors supplied semen samples that were then tested using a polymerase chain reaction (PCR) assay. While the exact diagnostic accuracy of this test for Ebola virus is not known, PCR assays are generally believed to have very high specificity (Lemmon and Gardner, 2008). In addition, 100% specificity is reported by Pinsky et al. (2015) for the Xpert Ebola Assay used in our motivating example and test results by Pettitt et al. (2017) have also confirmed near perfect specificity. Semen samples from EVD survivors were tested every 2 or 4 weeks (if a survivor had a positive test then the next test was scheduled in 2 weeks, otherwise in 4 weeks) beginning at least 8 months after EVD symptoms resolved. As a result of the varying testing schedules and other assumed to be non-EVD-related reasons, test results were sometimes unobserved at given time periods. These test results can be assumed to be missing at random, that is, not dependent on unobserved disease shedding status (although they clearly depend on observed shedding status and so are not missing completely at random). Generally speaking, the proportion of positive results declined over time. This decline can be modeled as either a declining prevalence of Ebola viral shedding, or a declining sensitivity to detect Ebola virus in a viral “shedder” where the decline is sensitivity is attributed to a decreasing viral load rather than a decrease in the effectiveness of the test. Only the second can be expressed as an LCM since there is only a single latent variable per participant rather than one latent variable per data point which would make the model unidentifiable.

Because of the known high specificity of the PCR assay, simpler, data-based approximations of an LCM could also be considered. We will propose two data-based indicator variables to approximate the unobserved disease status. If we expect the specificity of the test to be nearly or effectively 100%, it could be possible to assume all positives are true positives and simply classify those who had at least one positive test as a “shedder” and those without a positive test, a “nonshedder.” This reductive approach will break down with even extremely low rates of false positives, and also has bias associated with low sensitivity. However, we will show that modifications to this approach can allow for a more effective data-based indicator for shedding status which can perform as well as the maximum likelihood estimation (MLE) found using the EM algorithm for an LCM when specificity is high and an appropriate number of test results are observed.

There are several advantages to these simpler approximations despite their bias. First, most LCMs require full specification of a model for viral decay. This does not allow for easy modeling of nonmonotonic decay without the inclusion of additional parameters which could make the model unidentifiable. Our simpler approximations make no assumption about the shape of the decline in sensitivity. Second, standard R packages which can be used to implement the EM algorithm for binomial mixture models, such as flexmix (Grün and Leisch, 2008), make assumptions of conditional independence. This assumption, which is difficult to verify, means that given the “shedding” status of an individual, their test results are considered independent, and this assumption is known to bias results if it is violated (Albert and Dodd, 2004; Pepe and Janes, 2007). It is possible to implement the EM algorithm and include a correlation structure without the use of a standard package, as in Menten et al. (2008); Xu et al. (2013); Daggy et al. (2014). However, this is much more complex to implement and assumptions about the conditional dependence structure must be carefully justified which involves a deep understanding of the biological pathways of both disease status and diagnostic test (Pepe and Janes, 2007). Misspecification may not be detected by a lack-of-fit assessment and still result in substantial bias in estimation (Schofield et al., 2021). This can be especially limiting in the case of emerging infectious diseases. The simpler, nonparametric estimates, like those proposed in this paper, can relax assumptions of conditional independence without having to justify a correlation structure by considering each time point separately. Third, the use of the EM algorithm to find the MLE can lead to a couple of computational issues. For one, the EM algorithm is not computationally efficient and may converge slowly. In addition, it is only guaranteed to converge to a local maximum and may have difficulty converging at all if the parameters are on the boundary of the parameter space (Nettleton, 1999). This is of particular relevance given the high specificity of PCR-based assays which will make parameter estimates close to the boundary. Our simpler estimates do not employ the EM algorithm and thus do not suffer from these issues. In fact, without bias corrections, their estimation boils down to the calculation of simple sample proportions. This gives rise to our fourth advantage of simpler approximations: easy to implement sample size and power calculations.

This paper will expand on existing methods to estimate diagnostic accuracy and prevalence in the absence of a gold-standard test by modeling decaying sensitivity and introduce new approximation methods that can have several advantages in an applied setting. These methods will be specifically applicable in settings with repeated PCR assay test results or other diagnostic tests known to have high specificity, an approach commonly used to test for the persistence of a variety of infectious diseases over time.

## 2 | METHODS

### 2.1 | Latent class model

Let N equal the number of individuals who have been tested and let $\zeta_{i},\;i=1,\;\ldots \;,N$ represent the “shedding” status of each individual, where $\zeta_{i}=1$ indicates the individual exhibits prolonged viral shedding and $\zeta_{i}=0$ indicates the individual does not. Assume that

(1) $$\zeta_{i}∼Bernoulli(\theta ).$$

Notice that $\zeta_{i}$ does not depend on time which implies that participants who are labeled a “shedder” will always remain so and vice versa. Thus, it is important to note that the shedding status of an individual represents whether they at any time exhibited prolonged viral shedding since the commencement of the study, though their viral load may eventually decline to a level below detection via PCR assay. Let $y_{it}=1$ indicate a positive test result for person i at time t where each t indexes a 2-week period. Assume that

(2) $$y_{it}|\zeta_{i}=1∼Bernoulli\{\alpha (t)\},$$

(3) $$y_{it}|\zeta_{i}=0∼Bernoulli(\beta ).$$

Parameter β can be interpreted as the rate of false positives among the “nonshedders” and thus 1-β represents the test specificity. Function α(t) represents the detection probability among those whose viral shedding is prolonged, but continues to decline. Let T be the largest number of 2-week periods over which individuals were sampled and let $\Delta_{i}=\left(\delta_{i1},\;\ldots \;,\delta_{iT}\right)$ be a collection of indicator variables denoting whether a test was observed for person i at time t. We will assume that the distribution for $\Delta_{i}$ depends only on the observed $y_{it}$ and parameters other than θ,α, and β. This is reasonable assumption for our EVD application since the scheduled timing of the next visit depends on the most recent test result. Given this assumption, we obtain maximum likelihood estimates using only the observed test results, and unknown shedding status. The likelihood we wish to maximize is given in Equation (4).

(4) $$\begin{array}{c} L(\theta ,\alpha ,\beta |y,\zeta )=\prod_{i=1}^{N}{\left[\theta \prod_{t:\delta_{it}=1}\alpha {\left(t\right)}^{y_{it}}\{1-\alpha (t){\}}^{1-y_{it}}\right]}^{\zeta_{i}}\\ \;\times {\left[(1-\theta )\prod_{t:\delta_{it}=1}\beta^{y_{it}}(1-\beta {)}^{y_{it}}\right]}^{1-\zeta_{i}}. \end{array}$$

### 2.2 | EM algorithm

In order to allow for time dependence for α(t) and to limit the number of parameters we define the following:

(5) π=logit(θ),

(6) $$\alpha_{0}+\alpha_{1}t=logit\{\alpha (t)\},$$

(7) γ=logit(β).

The resulting $\Theta ={\left(\pi ,\alpha^{\prime },\gamma \right)}^{\prime }$ can be estimated using the EM algorithm via the R package flexmix. Let $t_{i}^{*}$ represent the time index of the first observed test for person, i. To initialize the algorithm, we assign those with $y_{it^{*}}=1$, an initial positive test, to have $\zeta_{i}=1$ and all others to have $\zeta_{i}=0$. Then let ${\hat{\beta }}^{\left(0\right)}$ be the sample proportion of the observed “nonshedder” data with positive tests and let ${\hat{\alpha }}^{\left(0\right)}$ be the coefficients from a logistic regression on the observed “shedder” data predicting positive tests by time point. Performing the “E-step” of this algorithm we find

(8) $$E(\zeta_{i}|\Theta ,y_{i}^{\left(obs\right)})=\frac{\theta \prod_{t:\delta_{it}=1}\alpha {\left(t\right)}^{y_{it}}\{1\;-\;\alpha (t){\}}^{1\;-\;y_{it}}}{\theta \prod_{t:\delta_{it}=1}\alpha {\left(t\right)}^{y_{it}}\{1\;-\;\alpha (t){\}}^{1\;-\;y_{it}}\;+\;(1\;-\;\theta )\beta^{\sum_{t:\delta_{it}=1}y_{it}}{\left(1\;-\;\beta \right)}^{\sum_{t:\delta_{it}=1}1-y_{it}}}.$$

Maximizing the resulting expected log-likelihood, $Q(\Theta |\Theta^{k})$, we have the following updates for the algorithm at iteration, k:

(9) $${\overset{ˆ}{\pi }}^{\left(k+1\right)}=logit\left\{\frac{\sum_{i=1}^{N}E(\zeta_{i}|\Theta^{\left(k\right)},\;y_{i}^{\left(obs\right)})}{N}\right\},$$

(10) $$\begin{array}{c} {\hat{\gamma }}^{\left(k+1\right)}=logit\left[\frac{\sum_{i=1}^{N}\sum_{t:\delta_{it=1}}y_{it}\left\{1\;-\;E(\zeta_{i}|\Theta^{\left(k\right)},\;y_{i}^{\left(obs\right)})\right\}}{\sum_{i=1}^{N}T_{i}\left\{1\;-\;E(\zeta_{i}|\Theta^{\left(k\right)},\;y_{i}^{\left(obs\right)})\right\}}\right] \end{array},$$

(11) $${\hat{\alpha }}^{\left(k+1\right)}=arg\;{max}_{\alpha }\;Q(\Theta |\Theta^{\left(k\right)}),$$

where $T_{i}=\sum_{t=1}^{T}\delta_{it}$. We can find ${\hat{\alpha }}^{\left(k+1\right)}$ numerically using standard generalized linear model (GLM) methods. The expectation and maximization steps are repeated until convergence. Standard errors for each coefficient can be calculated using the observed information from the Louis method (Louis, 1982). Let ${\hat{\theta }}_{MLE},\;\hat{\alpha }{\left(t\right)}_{MLE}$, and ${\hat{\beta }}_{MLE}$ represent the resulting estimates.

### 2.3 | Data-based approximations of latent variables

We propose two indicator variables, $z_{i}$ and $z_{i}^{*}$ meant to approximate $\zeta_{i}$.

(12) $$z_{i}=I\left(\exists \;t\;\text{s.t.}\;y_{it}=1\right),$$

(13) $$z_{i}^{*}=I\left(y_{it^{*}}=1\;\text{or}\;y_{it}=1\;\text{for}\;\text{multiple}\;t\right).$$

We hypothesize that these indicator variables may be useful to approximate $\zeta_{i}$ due to the high specificity of our test, thus we expect most, if not all, positive tests to come from those who are actually shedding the virus. Rationale for $z_{i}^{*}$ comes from the suspected decline in sensitivity to identify “shedders” over time, thus we are most likely to observe a positive from a “shedder” in their first test. Using $z_{i}$ or $z_{i}^{*}$ we can get estimates for $\Theta =(\theta ,\alpha ,\beta {)}^{\prime }$ using simple sample proportions. Assume that $z_{i}=1$ and $z_{i}=0$ for at least one person, i (and the same for $z_{i}^{*}$).

(14) $$\overset{ˆ}{\theta }=\frac{1}{N}\sum_{i=1}^{N}z_{i},$$

(15) $$\overset{ˆ}{\alpha }(t)=\frac{\sum_{i:\delta_{it}=1}z_{i}y_{it}}{\sum_{i:\delta_{it}=1}z_{i}},$$

(16) $$\overset{ˆ}{\beta }=\frac{\sum_{i=1}^{N}\sum_{t:\delta_{it}=1}\left(1\;-\;z_{i}\right)y_{it}}{\sum_{i=1}^{N}T_{i}\left(1\;-\;z_{i}\right)}=0,$$

(17) $${\overset{ˆ}{\theta }}^{*}=\frac{1}{N}\sum_{i=1}^{N}z_{i}^{*},$$

(18) $$\overset{ˆ}{\alpha }{\left(t\right)}^{*}=\frac{\sum_{i:\delta_{it}=1}z_{i}^{*}y_{it}}{\sum_{i:\delta_{it}=1}z_{i}^{*}},$$

(19) $${\overset{ˆ}{\beta }}^{*}=\frac{\sum_{i=1}^{N}\sum_{t:\delta_{it}=1}\left(1\;-\;z_{i}^{*}\right)y_{it}}{\sum_{i=1}^{N}T_{i}\left(1\;-\;z_{i}^{*}\right)}.$$

All of these estimates are biased to a degree. See the Web Appendix for the expectations for each estimate. Rearranging the expectation equations, we can also find expressions for bias corrections. Further details for these derivations can also be found in the Web Appendix. Let ${\hat{\theta }}_{BC}$ and ${\hat{\alpha }}_{BC}$ be the bias-corrected estimates for estimates found using $z_{i}$ and let ${\hat{\theta }}_{BC}^{*}$ and $\hat{\alpha }{\left(t\right)}_{BC}^{*}$ be the bias-corrected estimates for estimates found using $z_{i}^{*}$:

(20) $${\hat{\theta }}_{BC}=\frac{\overset{ˆ}{\theta }}{\frac{1}{N}\sum_{i=1}^{N}\left[1\;-\;\prod_{t:\delta_{it}=1}\{1\;-\;\overset{ˆ}{\alpha }(t)\}\right]},$$

(21) $$\overset{ˆ}{\alpha }{\left(t\right)}_{BC}=\frac{\overset{ˆ}{\alpha }\left(t\right)\left[1\;-\;\prod_{t:\delta_{it}=1}\left\{1\;-\;\overset{ˆ}{\alpha }\left(t\right)\right\}\right]}{{\overset{ˆ}{\theta }}_{BC}}.$$

Because $\hat{\beta }=0$, these estimates can correct for bias associated with $P\left(z_{i}=0|\zeta_{i}=1\right)>0$, but not for the bias associated with $P\left(z_{i}=1|\zeta_{i}=0\right)>0$.

Let $\psi_{it}(k)=P\left(z_{i}^{*}=1|y_{it}=1,\zeta_{i}=k\right)$, then

(22) $$\psi_{it}(k)=\left\{\begin{array}{ll} 1 & \;\text{if}\;t=t^{*}\\ \left\{\begin{array}{ll} 1-\prod_{j\neq t}\{1-\alpha (j)\} & \;\text{if}\;k=1\\ 1-{\left(1-\beta \right)}^{T_{i}-1} & \;\text{if}\;k=0 \end{array}\;t\neq t^{*},\right. \end{array}\right.$$

(23) θ^BC*=θ^*-1N∑i=1N1-∑j=01Ti-1jβ^*k1-β^*Ti-k1N∑i=1N1-∑j=01Ti-1jβ^*k1-β^*Ti-k-1N∑i=1N1-∏t:δit=11-α^(t)*-∑t:δit=1t≠t*α(t)∏k≠t1-α^(k)*,

(24) $$\overset{ˆ}{\alpha }{\left(t\right)}_{BC}^{*}=max\left(min\left[\frac{\overset{ˆ}{\alpha }{\left(t\right)}^{*}E({\overset{ˆ}{\theta }}^{*}|\Theta \;=\;{\overset{ˆ}{\Theta }}^{*})\;-\;\{1\;-\;{\overset{ˆ}{\theta }}_{BC}^{*}{\overset{ˆ}{\beta }}^{*}\frac{1}{N}\sum_{i=1}^{N}\psi_{it}(1)\}}{{\overset{ˆ}{\theta }}_{BC}^{*}\frac{1}{N}\sum_{i=1}^{N}\psi_{it}(1)},1-\epsilon \right],\epsilon \right).$$

Performing these bias corrections and substituting $\hat{\alpha }{\left(t\right)}^{*}$ for α(t) may result in values less than 0 or greater than 1. We thus take the minimum of the bias corrected expression and 1-ϵ and the maximum between the bias correction and ϵ where ϵ is a small positive number close to 0. Unlike the bias-corrected estimates from $z_{i}$, the biascorrected estimate using $z_{i}^{*}$ can correct for bias associated with $P\left(z_{i}=0|\zeta_{i}=1\right)>0$ and $P\left(z_{i}=1|\zeta_{i}=0\right)>0$.

#### 2.3.1 | Smoothing decay in sensitivity

Although $\overset{ˆ}{\alpha }(t),\overset{ˆ}{\alpha }{\left(t\right)}^{*},\;\overset{ˆ}{\alpha }{\left(t\right)}_{BC}$, and $\overset{ˆ}{\alpha }{\left(t\right)}_{BC}^{*}$ are calculated from sample means at each individual time point, we may still wish to represent the decline in test positivity among shedders as a smoothed curve. This can be done using cubic splines to create a trend line which is not constrained by parametric assumptions as strong as our MLE estimate. We will employ this smoothing technique to our motivating example of EVD survivors. We can also fit a logistic regression curve weighted by the sample size observed at each time point among “shedders.” We will consider the case of a simple logistic regression further when considering power calculations corresponding to the data-based indicator variables.

### 2.4 | Model comparisons

#### 2.4.1 | Bias, MSE, and coverage probability

To compare the operating characteristics of the five estimates (MLE using the EM algorithm, the sample proportions using $z_{i}$, the sample proportions using $z_{i}^{*}$, and the two bias-corrected estimates), it is useful to know the bias, mean squared error (MSE), and coverage probability of each under different circumstances. Bias and MSE can be determined analytically for the uncorrected sample proportions using the closed-form expressions for expected values calculated in the Web Appendix. Coverage probabilities and the MSE and bias for the other estimates will be calculated using the bootstrap.

#### 2.4.2 | Simulations

There are two major assumptions in our likelihood-based model: (1) conditional independence and (2) logistic decay in sensitivity. Our simulations will take place under three scenarios:

1. Conditional independence and logistic decay in sensitivity (both assumptions met);
2. Conditional dependence for “shedders” and logistic decay in sensitivity (first assumption met only);
3. Conditional independence and nonmonotonic decay in sensitivity (second assumption met only).

In all three scenarios, we simulate from a population with 30% of participants showing persistent viral shedding. Overall, sensitivity will decay in each simulation. Under scenarios 1 and 2, it will decay monotonically and in scenario 3 it will following a polynomial trend as specified in Equation (32). Plots of the assumed decay in sensitivity to detect “shedders” can be found in Web Figure 1. We assume that $\delta_{it}=1$ for all i and t, and, for each participant, the number of tests, T will vary, but the sensitivity of the first and last test will always be the same. Specificity will also be varied from 95% to 100%. The simulation parameters are described in Equations (25–32)

(25) n=100,

(26) T={3,4,5,6,7,8},

(27) θ=0.3,

(28) $$\beta =\left\{0,\;0.005,\;0.01,\;0.05\right\},$$

(29) $$\;\text{Scenario}\;1:\;\alpha (t)={logit}^{-1}\left\{2-\frac{3.5}{T\;-\;1}(t-1)\right\}\text{,}$$

(30) $$\;\text{Scenario}\;2:\;\alpha (t,i)={logit}^{-1}\left\{2-\frac{3.5}{T\;-\;1}(t-1)+\epsilon_{i}\right\}\text{,}\;$$

(31) $$\epsilon_{i}∼Normal(0,\;1),$$

(32) $$\;\text{Scenario}\;3:\;\alpha (t)=\left[{\left(\frac{t-1}{T-1}\right)}^{4}{\left(\frac{t-1}{T-1}\right)}^{3}\;\ldots \;1\right]\left[\begin{array}{c} -8.185\\ 15.214\\ -7.779\\ 0\\ 0.875 \end{array}\right]\text{.}$$

It should be noted that in the first scenario we are simulating data under the assumption that both the likelihood expressed in Equation (4) and the time dependency expressed in Equation (6) are correct. Thus, any bias or deviations in coverage probability for the MLE can be attributed to the possibility of the EM algorithm converging to a local maximum or small sample size rather than model misspecification. Scenarios 2 and 3 will illustrate ways in which the conditionally independent MLE may fail due to misspecification. For each T and β specification, we will run 1000 iterations and calculate each of the five estimates and their corresponding MSE, bias, and coverage probability.

#### 2.4.3 | Study design simulations

Using the simplest approximations, power calculations are fairly simple. Let Φ represent the cdf of the normal distribution and let $Z(\gamma )=\Phi^{-1}(\gamma )$. For the desired margin of error, d, and significance level, α, the following condition must be met:

(33) $$N\geq \frac{Z{\left(1\;-\;\alpha /2\right)}^{2}\theta (1\;-\;\theta )}{d^{2}}.$$

Because we have no estimate for θ before commencing the study, the most conservative estimate for sufficient sample size is found by assuming θ=0.5. According to Inequality 33, if we wish to have a margin of error of 5%, we would need a sample size of at least 385 participants.

We can use the Wald statistic for a simple logistic regression to perform a slightly more complex power calculation to determine the sample size needed to ensure enough power to show significant decay in sensitivity.

(34) $$\text{Power}=\sum_{k=0}^{N}P(\text{Wald}\;>Z(1-\alpha /2)|\sum \zeta_{i}=k)\;\;P(\sum \zeta_{i}=k)$$

(35) =∑k=0NΦα1XTWX22-1/k-Z(1-α/2)×Nkθk(1-θ)N-k.

$X_{T\times 2}$ is the fixed design matrix for a single participant and $W_{T\times T}=diag\{\frac{expa_{0}+a_{1}t}{{\left(1+expa_{0}+a_{1}t\right)}^{2}}\}$. This expression cannot be solved analytically, but can be plotted or solved numerically for N. For sufficiently small $\frac{a_{1}^{2}}{{\left(X^{T}WX\right)}_{22}^{-1}}$, the following can be used to determine N:

(36) $$N\theta \geq \frac{{\left\{Z(\text{Power})\;+\;Z(1\;-\;\alpha /2)\right\}}^{2}}{a_{1}^{2}/{\left(X^{T}WX\right)}_{22}^{-1}}.$$

If participants are not expected to be observed at every available time point, an estimate for missingness must also be considered and used to scale up the number of participants needed for the study. For example, if we only expect two-thirds of our participants to come in for a test at each time point, we would need to scale up the number of participants needed by a factor of 3/2. For simplicity, these simulations will assume that $N=N_{t},\forall t$, that is, that every individual is observed at each available testing time.

These power calculations assume that the sample proportions are unbiased estimates, which is not true in this case. We will simulate data using these sample size specifications to determine whether these power calculations can be useful.

To simulate the sufficient sample size to achieve an estimate for θ within a margin of error, d, we will simulate data under the following assumptions:

(37) N={100,200,300,385,400,500},

(38) T=8,

(39) θ=0.3,

(40) α(t)=logit(2-0.5t),

(41) β=0.01.

For each simulation we will find the resulting margin of error for each of the five estimates. Because most of our estimates are biased, we will also calculate the coverage probability to demonstrate when and if any of the estimates become too precise to capture the true value due to biasedness.

Second, we will simulate data to test whether enough power is achieved by each of our estimates to detect a difference between α(1) and α(t) using the following specifications:

(42) N={10,15,20,25,30},

(43) T=8,

(44) θ=0.3,

(45) α(t)=logit(2-0.5t),

(46) β=0.01.

For each sample size, we will calculate the expected power and the observed power for each of the estimates.

### 2.5 | Applications to Ebola virus data

To illustrate the use of these estimates, we will apply our methods to data on PCR assay results from semen samples from men who recovered from EVD in Liberia. We will then compare the results from each of the methods. We will also use the power calculations from Inequalities 33 and 35 as part of the design of a clinical trial of remdesivir in male survivors in the Democratic Republic of the Congo (DRC). This trial was motivated by the realization that male EVD survivors are capable of transmitting Ebola during sexual intercourse and the administration of remdesivir may reduce the frequency of viral shedding in male survivors. A similar trial was conducted in Liberia and Guinea but was stopped for futility due to an inability to identify male survivors who were still actively shedding. Many of the survivors in the DRC had been vaccinated or treated with more effective therapies than were available after the outbreak in West Africa and it has been hypothesized that the decay rate among these men is faster than among those who lacked access to vaccines or effective therapies.

## 3 | RESULTS

### 3.1 | Simulation results

**FIGURE 1:**
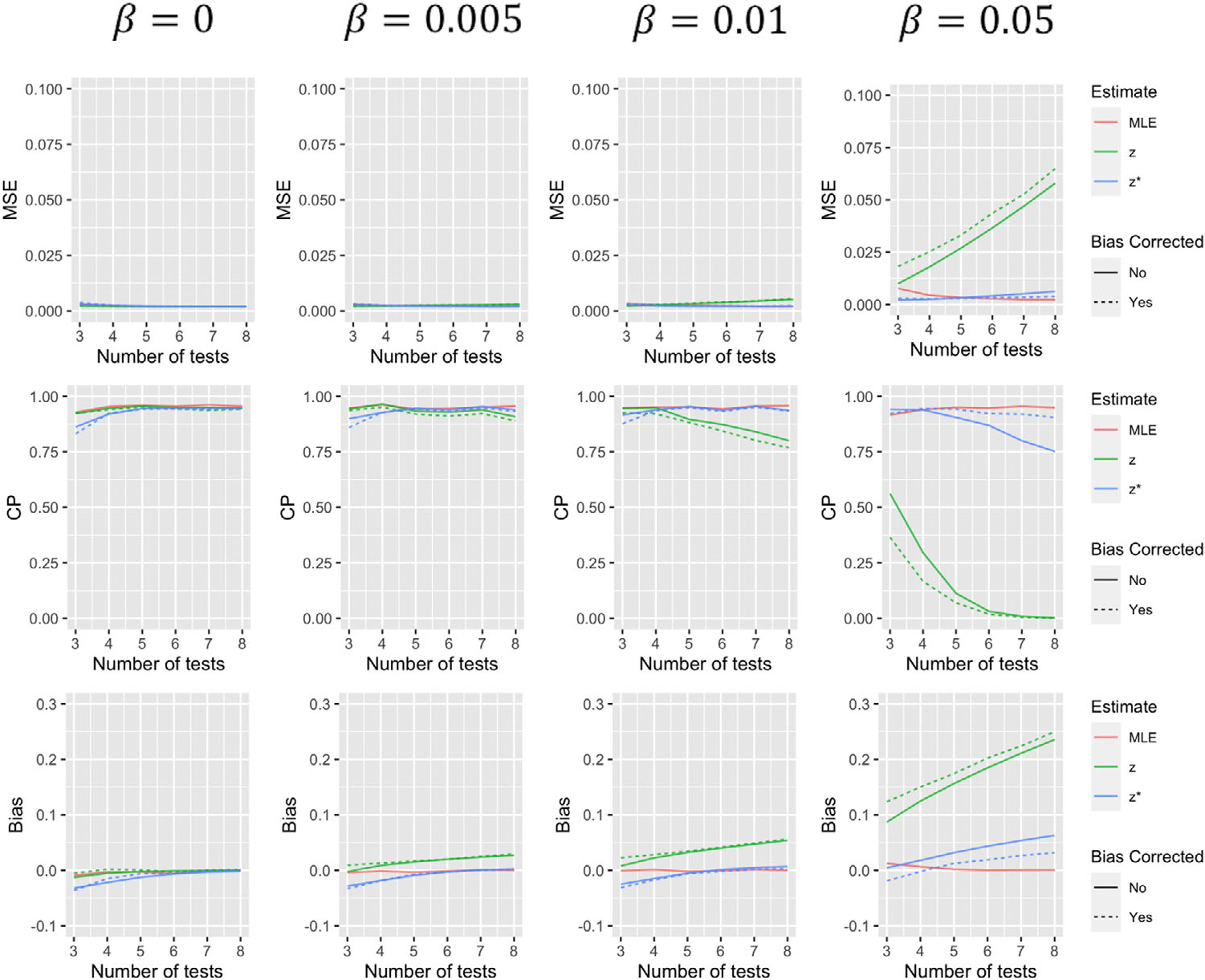
Simulation results for θ for scenario 1 summarizing mean squared error, coverage probability, and bias of the five estimates for different values of β and varying number of tests. This figure appears in color in the electronic version of this article, and any mention of color refers to that version

**FIGURE 2:**
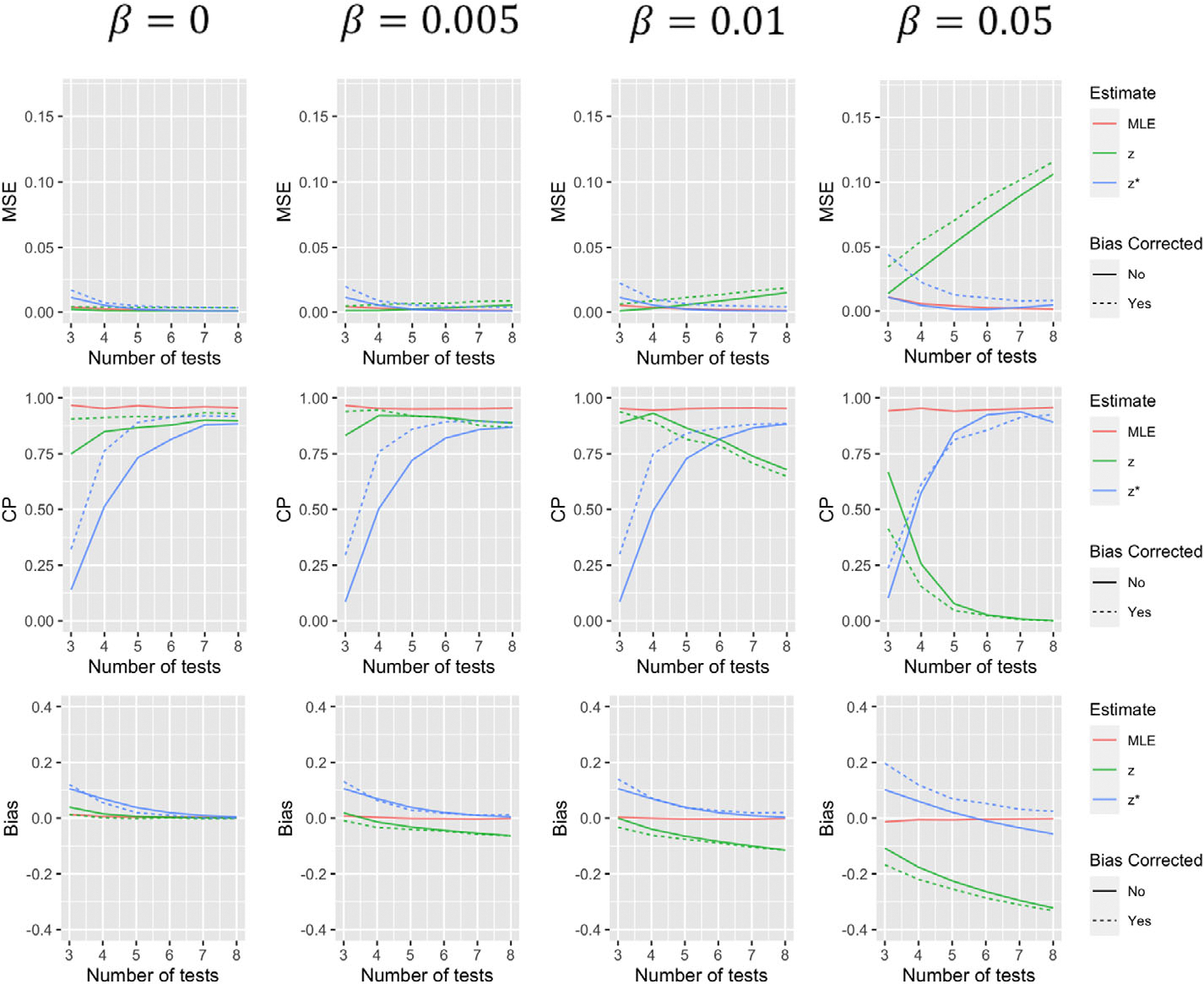
Simulation results for α(1) for scenario 1 summarizing mean squared error, coverage probability, and bias of the five estimates for different values of β and varying number of tests. This figure appears in color in the electronic version of this article, and any mention of color refers to that version

**FIGURE 3:**
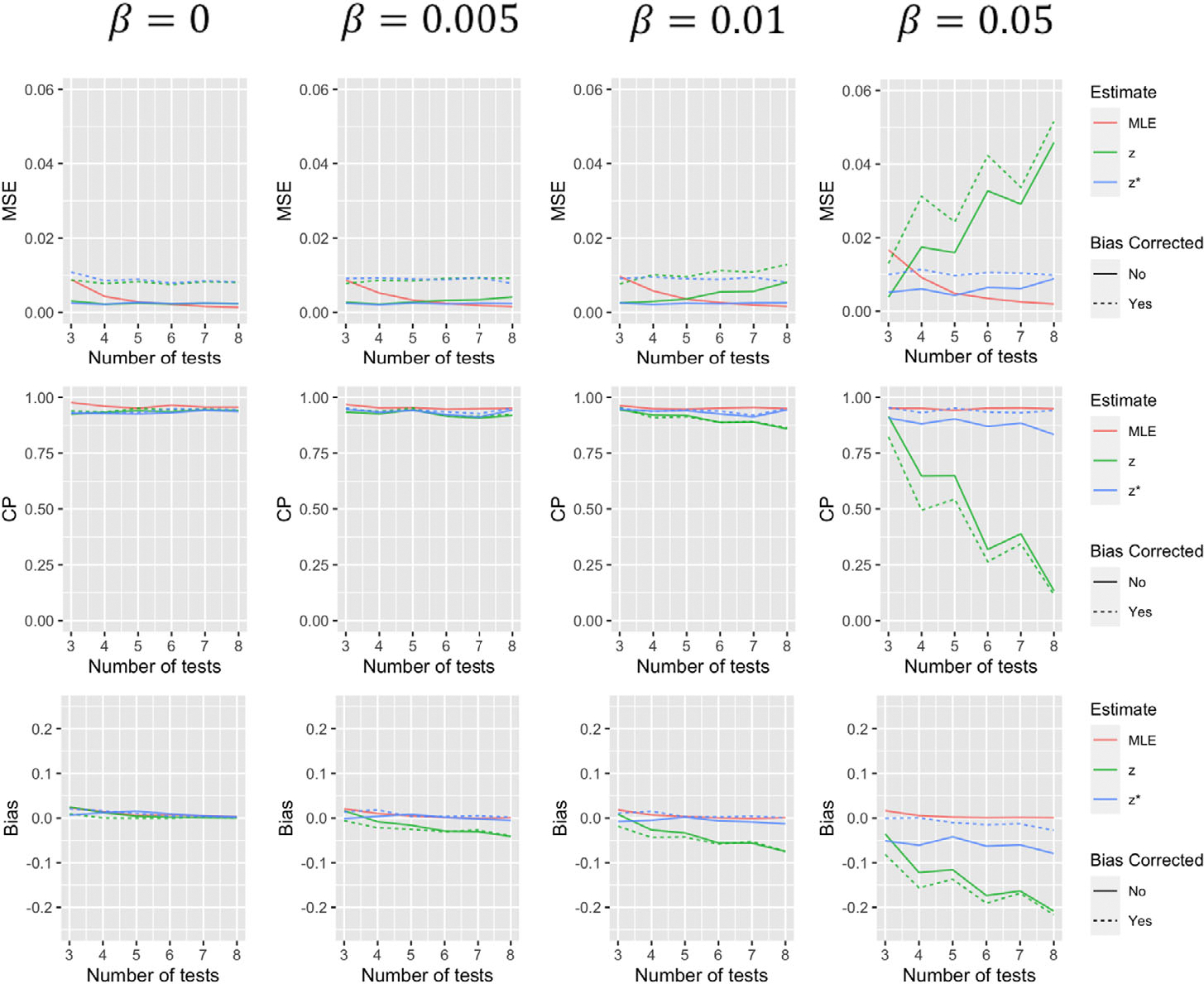
Simulation results for α(t) for scenario 1 where t is the median test summarizing mean squared error, coverage probability, and bias of the five estimates for different values of β and varying number of tests. This figure appears in color in the electronic version of this article, and any mention of color refers to that version

**FIGURE 4:**
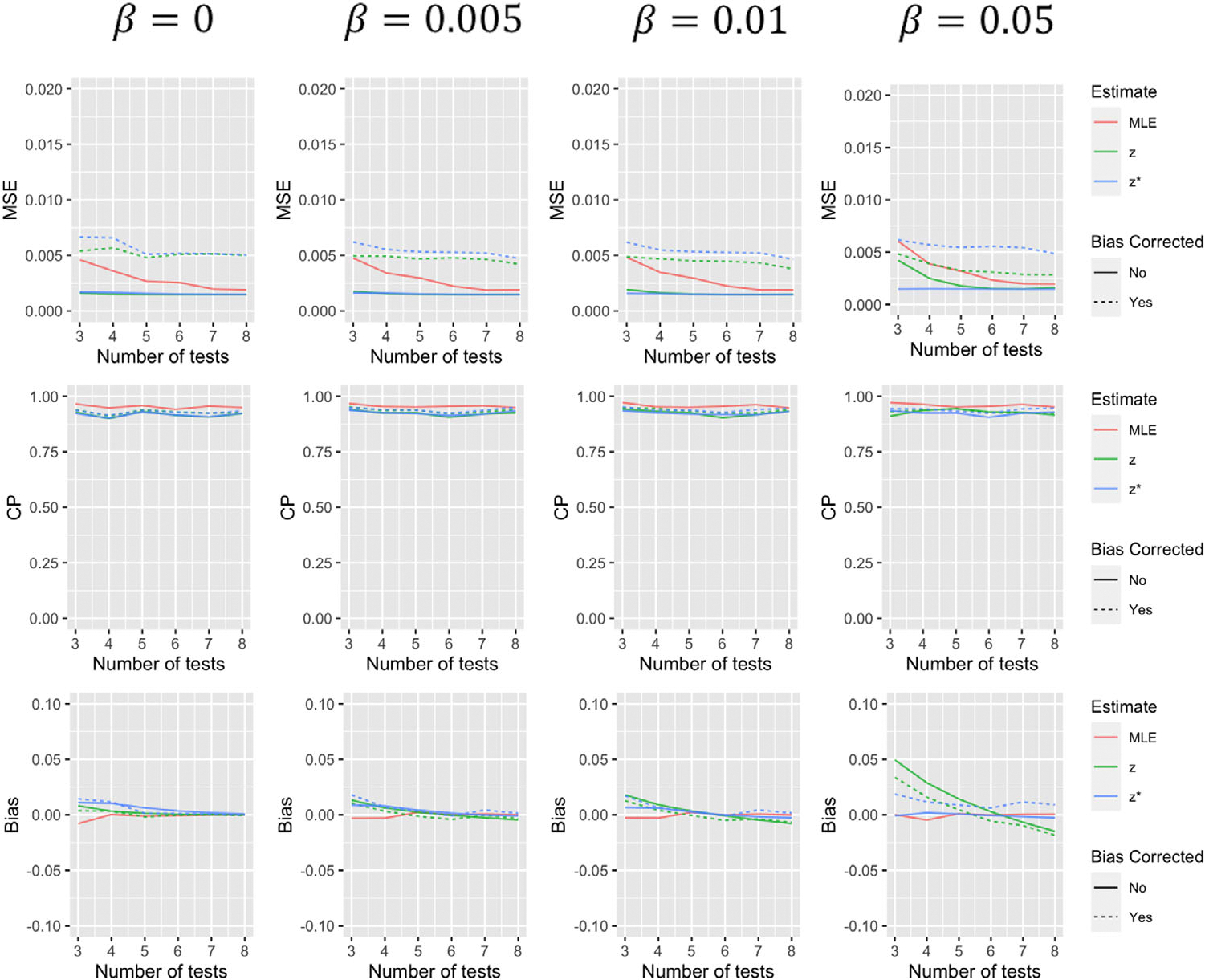
Simulation results for α(t)for scenario 1 summarizing mean squared error, coverage probability, and bias of the five estimates for different values of β and varying number of tests. This figure appears in color in the electronic version of this article, and any mention of color refers to that version

Simulation results comparing the operating characteristics of the five estimates for the first scenario are summarized in Figures 1–4. When β=0, that is, tests have 100% specificity, using $z_{i}$ to estimate $\zeta_{i}$ produces results with very similar MSE and coverage probability levels as the MLE. The bias-corrected version of this estimate effectively eliminates the small level of bias, but increases variance of the estimate resulting in larger MSE. As β increases, estimates using $z_{i}$ to approximate $\zeta_{i}$, with and without bias corrections, begin to suffer, especially when a greater number of tests are performed. The performance of estimates using $z_{i}^{*}$ to approximate $\zeta_{i}$, are less dependent on the value of β. With more tests, estimates using this approximation for θ tend to decline in performance, but much less dramatically than approximations using $z_{i}$. Furthermore, both the biascorrected and uncorrected versions of this approximation for α(t) tend to improve with more tests (to a point). While the bias correction tends to decrease bias and increase coverage probability of these estimates in most situations, it again tends to increase MSE. Estimates using $z_{i}^{*}$ to predict α(t), with and without a bias correction, tend to perform slightly worse when t=1 which makes some sense as all those with a positive initial test are classified as “shedders” under this approximation. It should be noted that α(t) estimates for other time points have coverage more similar to that of the median and final tests displayed in Figures 3 and 4.

Simulation results comparing the operating characteristics of the five estimates for the second scenario (conditionally dependent sensitivity) are summarized in Web Figures 2–5. Estimates for θ and α(1) show roughly the same trend as the previous scenario with slightly worse performance with our data-based estimates mostly noticeable when β is larger. At later time points, the MLE begins to suffer from increased bias and decreased coverage probability in comparison to our data-based estimates especially when β is low. Again we observe $z_{i}$ estimates to be useful only when β is approximately 0, but $z_{i}^{*}$ is more robust to lower specificity and even outperforms the MLE as the number of tests increases.

Simulation results comparing the operating characteristics of the five estimates for the third scenario (non-monotonic decay in sensitivity) are summarized in Web Figures 6– 9. Similar to the previous scenario, changing the functional form of α does little to affect the estimation of θ. However, the MLE for α(t) performs very poorly, especially when there are many tests, compared to the other estimates. Again we observe $z_{i}$ to be more useful with high specificity and fewer tests, and $z_{i}*$ to be more robust to lower specificity and improve with more tests.

In these scenarios, we did not observe any instances where the EM algorithm failed to converge. However, in addition to the three scenarios outlined in Equations (29–32), when we explored instances of lower shedding prevalence (θ=0.1) we did find that the EM algorithm failed to identify a single “shedder” 2.9–14.7% of the time when n=100 (varying with the degree of tests and false positive rate) and up to 4.2% when n=300. We did not observe these failures to obtain estimates with $z_{i}$ or $z_{i}^{*}$, bias corrected or otherwise. We also experimented with other model misspecifications that violate both assumptions of conditional independence and logistic decay and found similar overall trends in decreased relative performance of the MLE, although they were not as dramatic as those observed in scenarios 2 and 3. This could likely be attributed to both monotonic (although not logistic decay) and a lower level of overall conditional dependence.

### 3.2 | Study design simulation results

Plots summarizing the results of the study design simulations can be found in the Supporting Information. Using Inequality 33, we determined that a sample size of 385 would be necessary to achieve a margin of error of 0.05. Web Figure 10 shows the expected power using Inequality 33 as a dashed line. Although not all estimates are simple sample proportions, the margin of error is not much different for each of the estimates. The coverage probability for the MLE, $z^{*}$, and $z^{*}$ with a bias correction stays consistently around 0.95 regardless of sample size. The coverage probability using z, with or without a correction, decreases as the sample size grows. This finding is consistent with earlier simulation results when β=0.01.

Web Figure 11 shows that power calculations assuming simple logistic regression can be used to inform the appropriate sample size for all five estimates. The dashed line represents the expected power given Inequality 35. In fact, $z_{i}^{*}$, shows higher than expected power more similar to the LCM results than those expected for a simple logistic regression.

### 3.3 | Results using PREVAIL III data

We calculated each of the proposed estimates on data collected from a cohort of survivors from the 2013–2016 West African EVD outbreak. Overall, there were N=265 participants with at least one test result. Tests were administered at a schedule of every 2 or 4 weeks, thus each time point represents a 2-week interval. The first testing time point occurred around 8 months after EVD symptoms resolved. In total there were T=50 possible time points, but on aver age only 8.8 test results were observed. Unobserved test results are assumed to be missing at random, therefore we can ignore the missingness process.

We anticipate the results from Section 3.1 with eight tests and β=0.01 to be the most informative for the performance of approximations in our case. Using the MLE estimate, we find that around 33.9% (95% CI: 24.2%, 43.5%) of participants exhibited persistent viral shedding in semen 8 months after acute disease symptoms resolved.

(47) $$\overset{ˆ}{\alpha }(t)=\frac{exp\left({\overset{ˆ}{\alpha }}_{0}\;+\;{\overset{ˆ}{\alpha }}_{1}t\right)}{1\;+\;exp\left({\overset{ˆ}{\alpha }}_{0}\;+\;{\overset{ˆ}{\alpha }}_{1}t\right)},$$

(48) $${\overset{ˆ}{\alpha }}_{0}=2.13\;(95\%CI:1.394,\;2.865),$$

(49) $${\overset{ˆ}{\alpha }}_{1}=-0.139\;(95\%CI:-0.168,-0.109).$$

The sensitivity to detect Ebola virus in a persistent “shedder” at t=1 or 8 months after symptoms resolve is 88%. The odds of detecting Ebola virus in a persistent “shedder” declines 1.15 times with each passing 2-week interval of time. The effect of time is statistically significant. Table 1 compares all five estimates for $\theta ,\alpha \left(t\right),t=1,\;11,\;21,\;31$, and β.

**FIGURE 5:**
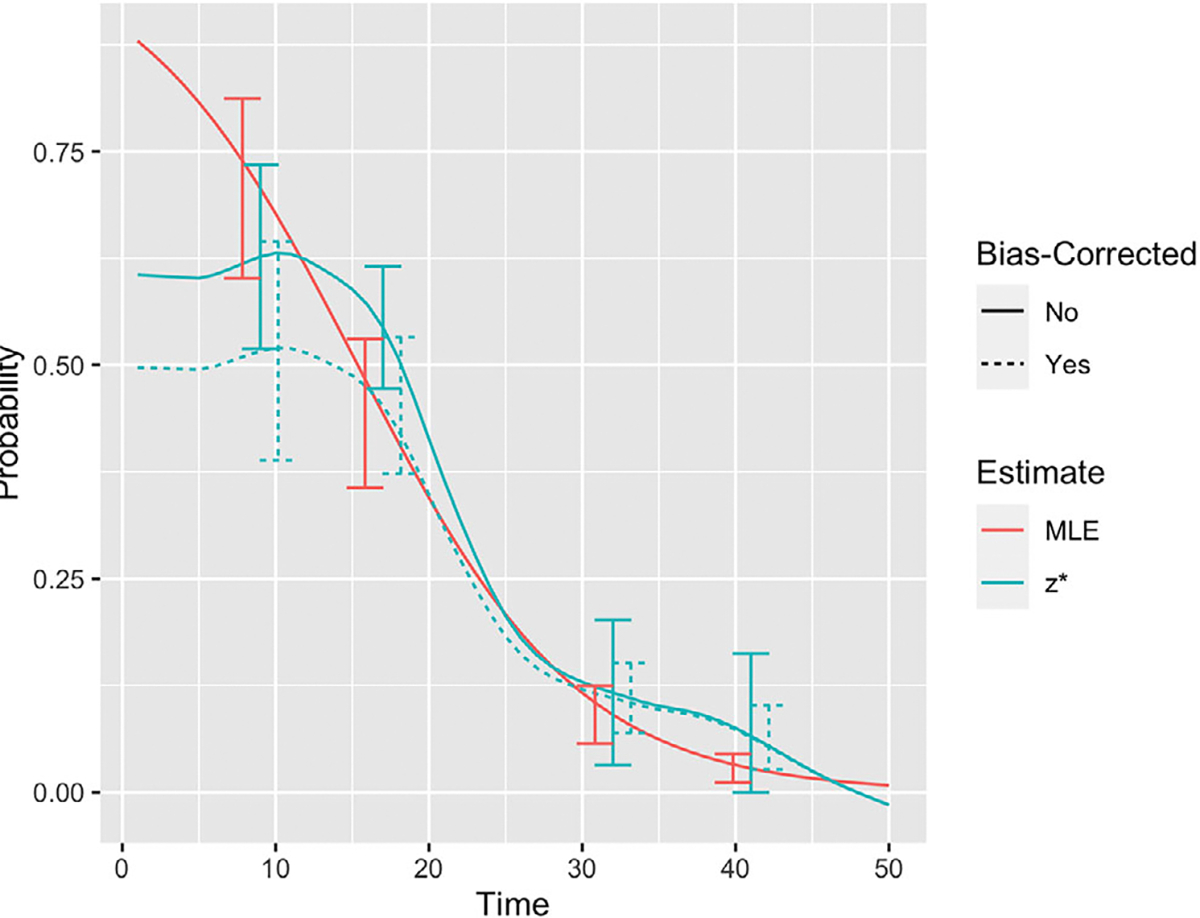
Sensitivity to detect “shedders” using the $\hat{\alpha }(t{)}_{MLE}$, and $\hat{\alpha }{\left(t\right)}^{*}$, and $\hat{\alpha }{\left(t\right)}_{BC}^{*}$ smoothed using cubic splines. This figure appears in color in the electronic version of this article, and any mention of color refers to that version

The estimates for θ range from 0.211 to 0.415. All four of the approximation confidence intervals overlap with the MLE’s confidence interval. All estimates show a declining sensitivity to detect Ebola virus in “shedders.” The sample proportion estimates using both $z_{i}$ and $z_{i}^{*}$ estimate the sensitivity to be exactly 1 at t=1. It should be noted that the data are very sparse at this time point (only one individual was tested this early, and he had a positive result). All the confidence intervals using approximations overlap with the MLE’s confidence interval. Many of these time points have only a handful of observations and thus the confidence intervals for α(t) are quite wide. Based on simulation results, we expected the MLE, $z_{i}^{*}$, and $z_{i}^{*}$ with a bias correction to perform the best for this example. Figure 5 shows the trend for α(t) using these estimates. The approximations are smoothed by fitting a weighted logistic regression.

Again we see overlapping confidence intervals for the three estimates showing they are not significantly different at the 95% confidence level.

### 3.4 | Calculating sample size for a follow-up study

We can recommend sample sizes for a similar study on Ebola virus persistence that was planned to take place in the DRC. For the proportion of “shedders,” if we assume $\theta ={\hat{\theta }}_{MLE}$ the recommended sample sizes for a margin of error of d=0.01,0.05,0.1 are 8608, 345, and 97, respectively. More conservatively, assuming θ=0.5, the recommended sample sizes to achieve the same margin of error would be 9604, 385, and 97, respectively.

For a similar study, where participants are tested every 2 weeks, we can make recommendations for the number of participants and number of tests needed to have sufficient power to detect a significant decline in sensitivity. Overall, we find that to achieve 80% power with 3, 5, or 7 tests, we would need 5173, 946, and 312 participants, respectively. This is summarized in greater detail in the first three rows of Web Table 1. We could also test the hypothesis that the change in sensitivity in the DRC for “shedders” is different than what was observed in Liberia. Assuming the decline in the DRC is 1.5 times as fast, to achieve 80% power for 3, 5, or 7 tests we would need 397, 153, and 34 participants, respectively. The second half of Web Table 1 summarizes these results in greater detail. These values were calculated using the following power expression:

(50) Power=∑k=0NΦa^1-log(1.5)-a^1XTWX22-1/k+Var^a^1-Z(0.975)×nkθk(1-θ)n-k.

When participants are not expected to be observed at every time point, sample sizes must be scaled up to account for this loss.

## 4 | DISCUSSION

When a gold-standard test is absent, an LCM can be used to represent the unobservable patient status. In a case where the test sensitivity is dependent on time, but the specificity remains constant, the EM algorithm can be implemented, but it is only guaranteed to converge to a local maximum and may become unidentifiable with increased flexibility. For tests with high specificity, such as PCR assays, data-based approximations can be considered. We introduced two approximations for the true status of patient, $i:\;z_{i}$ and $z_{i}^{*}$. While the more obvious $z_{i}$ proved to be too simplistic to be useful outside of the scenario where specificity is 100%, a slight modification of this approach, $z_{i}^{*}$, yields results on par with the MLE in many scenarios. These nonparametric, data-based approximations were also observed to be more robust to violations of the likelihood assumptions such as conditional independence and logistic decay in sensitivity. Bias-correction attempts, were able to improve the coverage probability and decrease bias when estimating the proportion of persistent “shedders,” but in some scenarios increased MSE especially when estimating the sensitivity for “shedders” over time.

Sample size calculations are very straightforward for sample proportions and simple logistic regression. Designing a study with sufficient sample size to obtain precise proportion estimates and enough power to demonstrate a significant decline in sensitivity can easily be done for simple approximations. Furthermore, these sample size calculations assuming a simple model also prove useful for the more complicated bias-corrected and EM algorithm estimates.

In an application of these estimates for data on persistence of Ebola virus in semen of men after recovery from EVD, we found estimates for the proportion with persistent shedding, the changing sensitivity of the PCR assay over time and the specificity of the assay. From the MLE found via the EM algorithm and the $z_{i}^{*}$ approximations, we found that around a fifth to a third of all men exhibited persistent viral shedding in semen 8 months after recovery from acute EVD symptoms. Of these persistent shedders, the sensitivity to continue to detect Ebola viral shedding in semen declined over the course of the study. This decline in sensitivity implies a decrease in the viral load below detectable levels for the PCR assay. Our results show a high specificity of over 95% for all estimates and over 98% using the MLE which is consistent with the previous studies showing high specificity of this PCR assay. Although this method can help us understand when viral loads of “shedders” drop below detectable levels, incorporating continuous measurements of viral load among “shedders” over time could give a more precise and useful understanding of how the virus declines among “shedders.” This remains a topic for future studies.

These estimation methods could be useful for the design of future studies on Ebola virus persistence in semen such as the cohort study planned in the DRC mentioned previously. Beyond Ebola virus, many other viruses are detected using high specificity PCR assays, for example, SARS-CoV-2. There are still many unanswered questions about viral persistence after recovery from COVID-19 (Qi et al., 2020) and estimation methods such as these could inform power calculations for a cohort study and could also be employed to analyze the prevalence and sensitivity to detect persistent viral shedding over time.

## Supplementary Material

Drew_Biometrics_2023_supp

Drew_Biometrics_2023_zip

Drew_Biometrics_2023_github

SUPPORTING INFORMATION

The Web Appendix referenced in Sections 2.3 and 2.4, Web Figures referenced in Sections 2.4.2, 3.1, 3.2 and 3.4 and the Web Table referenced in 3.4 are available with this paper at the Biometrics website on Wiley Online Library. The simulation R code is available both on Wiley Online Library and on Github: https://github.com/clara-drew/LCM-simplification.

## Figures and Tables

**TABLE 1:** Estimates using PREVAIL-III data on shedding Ebola virus in semen

	*θ*	*α* (1)	*α* (11)	*α* (21)	*α* (31)	*β*

MLE	0.339 (0.242, 0.436)	0.88 (0.804, 0.955)	0.646 (0.54, 0.752)	0.314 (0.246, 0.382)	0.103 (0.067, 0.139)	0.011 (0.003, 0.019)
*z_i_*	0.302 (0.247, 0.361)	1 (0.025, 1)	0.667 (0.384, 0.882)	0.226 (0.096, 0.411)	0.067 (0.002, 0.319)	0
$z_{i}^{*}$	0.211 (0.164, 0.265)	1 (0.025, 1)	0.75 (0.428, 0.945)	0.227 (0.078, 0.454)	0.077 (0.002, 0.36)	0.014 (0.009, 0.02)
*z_i_*-BC	0.415 (0.338, 0.486)	0.728 (0.655, 0.793)	0.485 (0.295, 0.674)	0.164 (0.059, 0.281)	0.049 (0, 0.162)	
$z_{i}^{*}$-BC	0.257 (0.196, 0.33)	0.822 (0.742, 0.908)	0.618 (0.379, 0.832)	0.192 (0.049, 0.348)	0.072 (0, 0.239)	0.021 (0.003, 0.044)

## Data Availability

The data that support the findings of this study are available on request from the corresponding author. The data are not publicly available due to privacy or ethical restrictions.
