# Supplementary material for: Simplifying the estimation of diagnostic testing accuracy over time for high specificity tests in the absence of a gold standard
Source: Biometrics. Author manuscript; Available in PMC 2026 May 18. (PMC13181389; doi:10.1111/biom.13689)
Supplement: Drew_Biometrics_2023_supp [file NIHMS2149336-supplement-Drew_Biometrics_2023_supp.pdf]

Supporting Information for Simplifying the Estimation of Diagnostic Testing Accuracy Over Time for High Specificity Tests in the Absence of a Gold Standard

by

Clara Drew, Moses Badio, Dehkontee Dennis, Lisa Hensley,
Elizabeth Higgs, Michael Sneller, Mosoka Fallah, Cavan Reilly

1 Web Appendix

$$
\begin{aligned}
\mathrm{E}[\widehat{\theta}] &= \frac{1}{N}\sum_{i=1}^{N} P(z_i = 1) \\
&= \frac{1}{N}\sum_{i=1}^{N} P(z_i = 1|\zeta_i = 1)P(\zeta_i = 1) + P(z_i = 1|\zeta_i = 0)P(\zeta_i = 0) \\
&= \theta\frac{1}{N}\sum_{i=1}^{N}\Big[1 - \prod_{t:\delta_{it}=1}\big\{1-\alpha(t)\big\}\Big] + (1-\theta)\frac{1}{N}\sum_{i=1}^{N}\Big\{1 - \big(1-\beta\big)^{T_i}\Big\}. \qquad (1)
\end{aligned}
$$

Note that $P(z_i = 1|y_{it} = 1) = 1$

$$
\begin{aligned}
\mathrm{E}\{\widehat{\alpha}(t)\} &= \frac{\sum_{i=1}^{N} P(z_i = 1|y_{it} = 1, \zeta_i = 1)P(y_{it} = 1|\zeta_i = 1)P(\zeta_i = 1) + P(z_i = 1|y_{it} = 1, \zeta_i = 0)P(y_{it} = 1|\zeta_i = 0)P(\zeta_i = 0)}{\sum_{i=1}^{N} P(z_i = 1|\zeta_i = 1)P(\zeta_i = 1) + P(z_i = 1|\zeta_i = 0)P(\zeta_i = 0)} \\
&= \frac{\theta\alpha(t) + (1-\theta)\beta}{\theta\frac{1}{N}\sum_{i=1}^{N}\Big[1 - \prod_{t:\delta_{it}=1}\big\{1-\alpha(t)\big\}\Big] + (1-\theta)\frac{1}{N}\sum_{i=1}^{N}\Big\{1-\big(1-\beta\big)^{T_i}\Big\}}. \qquad (2)
\end{aligned}
$$

Solving equations 1 and 2 for θ and $\alpha(t)$ respectively we can get bias-corrected expressions:

$$
\begin{aligned}
\theta &= \frac{\mathrm{E}[\widehat{\theta}] - \frac{1}{N}\sum_{i=1}^{N}\{1-(1-\beta)^{T_i}\}}{\frac{1}{N}\sum_{i=1}^{N}\Big[1 - \prod_{t:\delta_{it}=1}\big\{1-\alpha(t)\big\}\Big] - \frac{1}{N}\sum_{i=1}^{N}\Big\{1-\big(1-\beta\big)^{T_i}\Big\}}, \\
\alpha(t) &= \frac{\mathrm{E}\{\widehat{\alpha}(t)\}\bigg(\Big[1 - \prod_{t:\delta_{it}=1}\big\{1-\alpha(t)\big\}\Big] + (1-\theta)\frac{1}{N}\sum_{i=1}^{N}\Big\{1-\big(1-\beta\big)^{T_i}\Big\}\bigg) - (1-\theta)\beta}{\theta}.
\end{aligned}
$$

Using these expressions we can define bias-corrected estimates for θ and $\alpha(t)$. Recall that $\widehat{\beta} = 0$.

$$
\begin{aligned}
\widehat{\theta}_{BC} &= \frac{\widehat{\theta}}{\frac{1}{N}\sum_{i=1}^{N}\big[1 - \prod_{t:\delta_{it}=1}\{1-\widehat{\alpha}(t)\}\big]}, \\
\widehat{\alpha}(t)_{BC} &= \frac{\widehat{\alpha}(t)\Big[1 - \prod_{t:\delta_{it}=1}\{1-\widehat{\alpha}(t)\}\Big]}{\widehat{\theta}_{BC}}.
\end{aligned}
$$

Because $\widehat{\beta} = 0$, these estimates can correct for bias associated with $P(z_i = 0|\zeta_i = 1) < 1$, but not for the bias associated with $P(z_i = 1|\zeta_i = 0) > 0$.
Now let's consider estimates using z_i^*.

$$
\begin{aligned}
\mathrm{E}(\widehat{\theta}^*) &= \frac{1}{N}\sum_{i=1}^{N} P(z_i = 1) \\
&= \frac{1}{N}\sum_{i=1}^{N} P(z_i^* = 1|\zeta_i = 1)P(\zeta_i = 1) + P(z_i^* = 1|\zeta_i = 0)P(\zeta_i = 0) \\
&= \theta\frac{1}{N}\Big[1 - \prod_{t:\delta_{it}=1}\{1-\alpha(t)\} - \sum_{\substack{t:\delta_{it}=1 \\ t\neq t^*}}\alpha(t)\prod_{k\neq t}\{1-\alpha(k)\}\Big] + (1-\theta)\frac{1}{N}\sum_{i=1}^{N}\Big\{1 - \sum_{j=0}^{1}\binom{T_i - 1}{j}\beta^j\big(1-\beta\big)^{T_i-j}\Big\},
\end{aligned}
$$

$$
\mathrm{E}\{\widehat{\alpha}(t)^*\} = \frac{\sum_{i=1}^{N} P(z_i^* = 1|y_{it} = 1, \zeta_i = 1)P(y_{it} = 1|\zeta_i = 1)P(\zeta_i = 1) + P(z_i^* = 1|y_{it} = 1, \zeta_i = 0)P(y_{it} = 1|\zeta_i = 0)P(\zeta_i = 0)}{\sum_{i=1}^{N} P(z_i^* = 1|\zeta_i = 1)P(\zeta_i = 1) + P(z_i^* = 1|\zeta_i = 0)P(\zeta_i = 0)}.
$$

Let $\psi_{it}(k) = P(z_i^* = 1|y_{it} = 1, \zeta_i = k)$, then

$$\psi_{it}(k) = \begin{cases} 1 & \text{if } t = t^* \\ \begin{cases} 1 - \prod_{j \neq t} \left\{1 - \alpha(j)\right\} & \text{if } k = 1 \\ 1 - \left(1 - \beta\right)^{T_i - 1} & \text{if } k = 0 \end{cases} & t \neq t^* \end{cases},$$

$$\begin{aligned}
\mathrm{E}\{\widehat{\alpha}(t)^*\} &= \frac{\theta\alpha(t)\frac{1}{N}\sum\limits_{i=1}^{N}\psi_{it}(1) + (1-\theta)\beta\frac{1}{N}\sum\limits_{i=1}^{N}\psi_{it}(0)}{\theta\frac{1}{N}\sum\limits_{i=1}^{N}\left[1 - \prod\limits_{t:\delta_{it}=1}\left\{1-\alpha(t)\right\} - \sum\limits_{\substack{t:\delta_{it}=1\\ t\neq t^*}}\alpha(t)\prod\limits_{k\neq t}\left\{1-\alpha(k)\right\}\right] - (1-\theta)\frac{1}{N}\sum\limits_{i=1}^{N}\left\{1 - \sum\limits_{j=0}^{1}\binom{T_i-1}{j}\beta^j\left(1-\beta\right)^{T_i-j}\right\}} \\
&= \frac{\theta\alpha(t)\frac{1}{N}\sum\limits_{i=1}^{N}\psi_{it}(1) + (1-\theta)\beta\frac{1}{N}\sum\limits_{i=1}^{N}\psi_{it}(0)}{\mathrm{E}(\widehat{\theta}^*)}.
\end{aligned}$$

Again we can get bias-corrected estimates by rearranging these expressions and substituting in sample proportion estimates where needed.

$$\begin{aligned}
\widehat{\theta}^*_{BC} &= \frac{\widehat{\theta}^* - \frac{1}{N}\sum\limits_{i=1}^{N}\left\{1 - \sum\limits_{j=0}^{1}\binom{T_i-1}{j}\widehat{\beta}^{*k}\left(1-\widehat{\beta}^*\right)^{T_i-k}\right\}}{\frac{1}{N}\sum\limits_{i=1}^{N}\left\{1 - \sum\limits_{j=0}^{1}\binom{T_i-1}{j}\widehat{\beta}^{*k}\left(1-\widehat{\beta}^*\right)^{T_i-k}\right\} - \frac{1}{N}\sum\limits_{i=1}^{N}\left[1 - \prod\limits_{t:\delta_{it}=1}\left\{1-\widehat{\alpha}(t)^*\right\} - \sum\limits_{\substack{t:\delta_{it}=1\\ t\neq t^*}}\alpha(t)\prod\limits_{k\neq t}\left\{1-\widehat{\alpha}(k)^*\right\}\right]}, \\
\widehat{\alpha}(t)^*_{BC} &= \max\left(\min\left[\frac{\widehat{\alpha}(t)^*\mathrm{E}(\widehat{\theta}^*|\Theta = \widehat{\Theta}^*) - \left\{1 - \widehat{\theta}^*_{BC}\widehat{\beta}^*\frac{1}{N}\sum_{i=1}^{N}\psi_{it}(1)\right\}}{\widehat{\theta}^*_{BC}\frac{1}{N}\sum_{i=1}^{N}\psi_{it}(1)},\ 1-\epsilon\right],\ \epsilon\right).
\end{aligned}$$

H

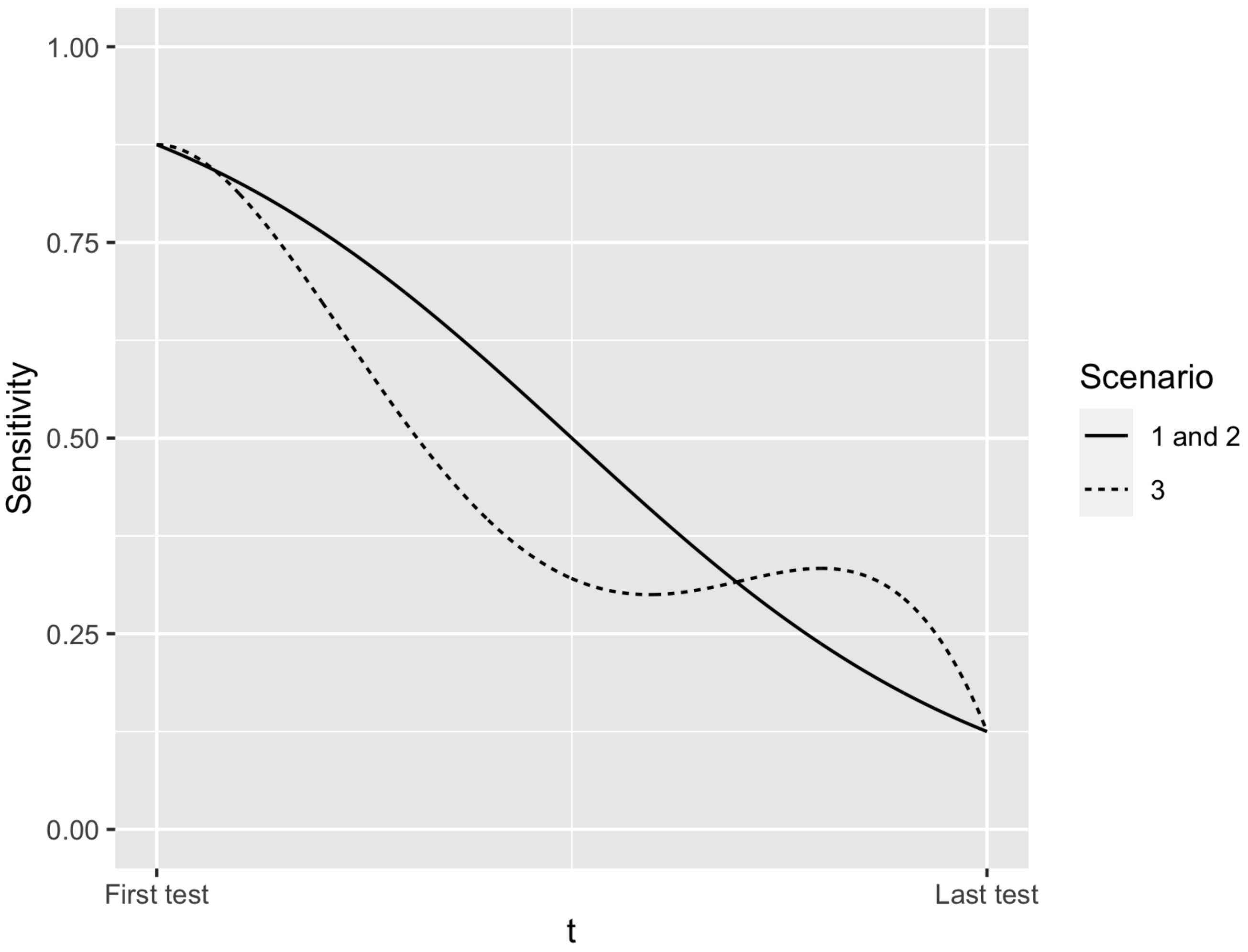

Web Figure 1: Assumed trend in decaying sensitivity to detect "shedders" for each simulation scenario

2 Web Figures

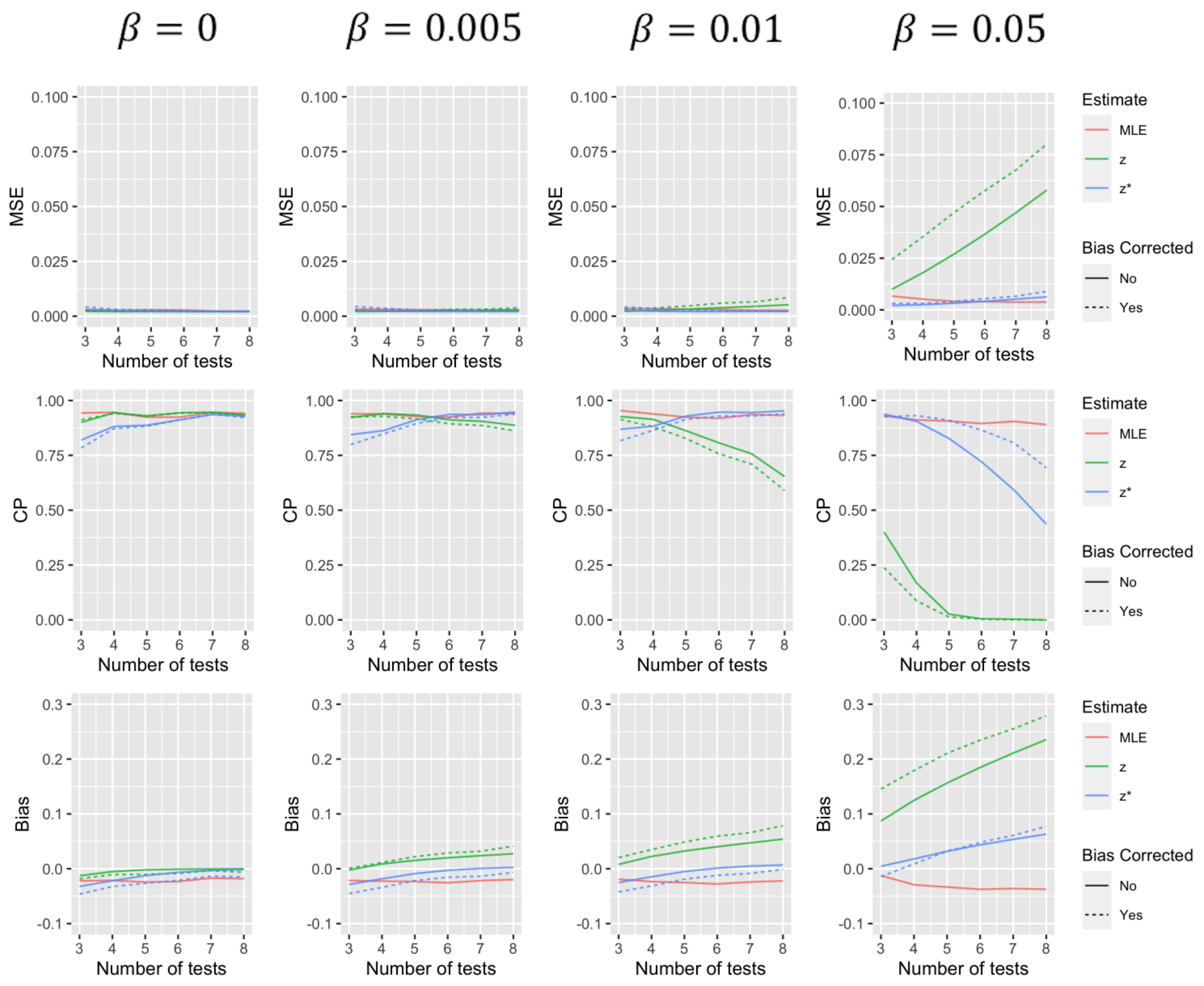

Web Figure 2: Simulation results for θ for scenario 2 summarizing mean squared error, coverage probability and bias of the five estimates for different values of β and varying number of tests.

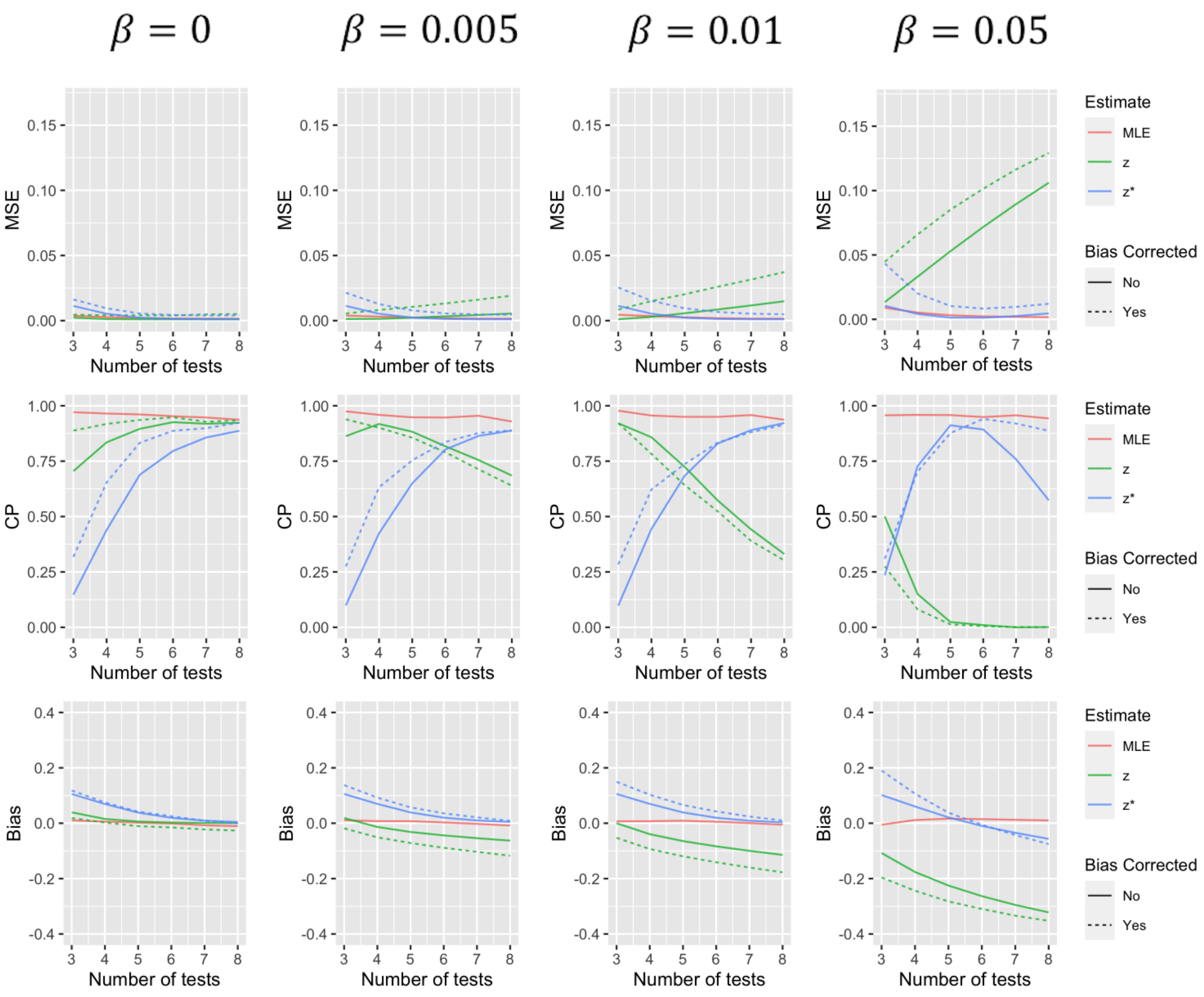

Web Figure 3: Simulation results for $\alpha(1)$ for scenario 2 summarizing mean squared error, coverage probability and bias of the five estimates for different values of β and varying number of tests.

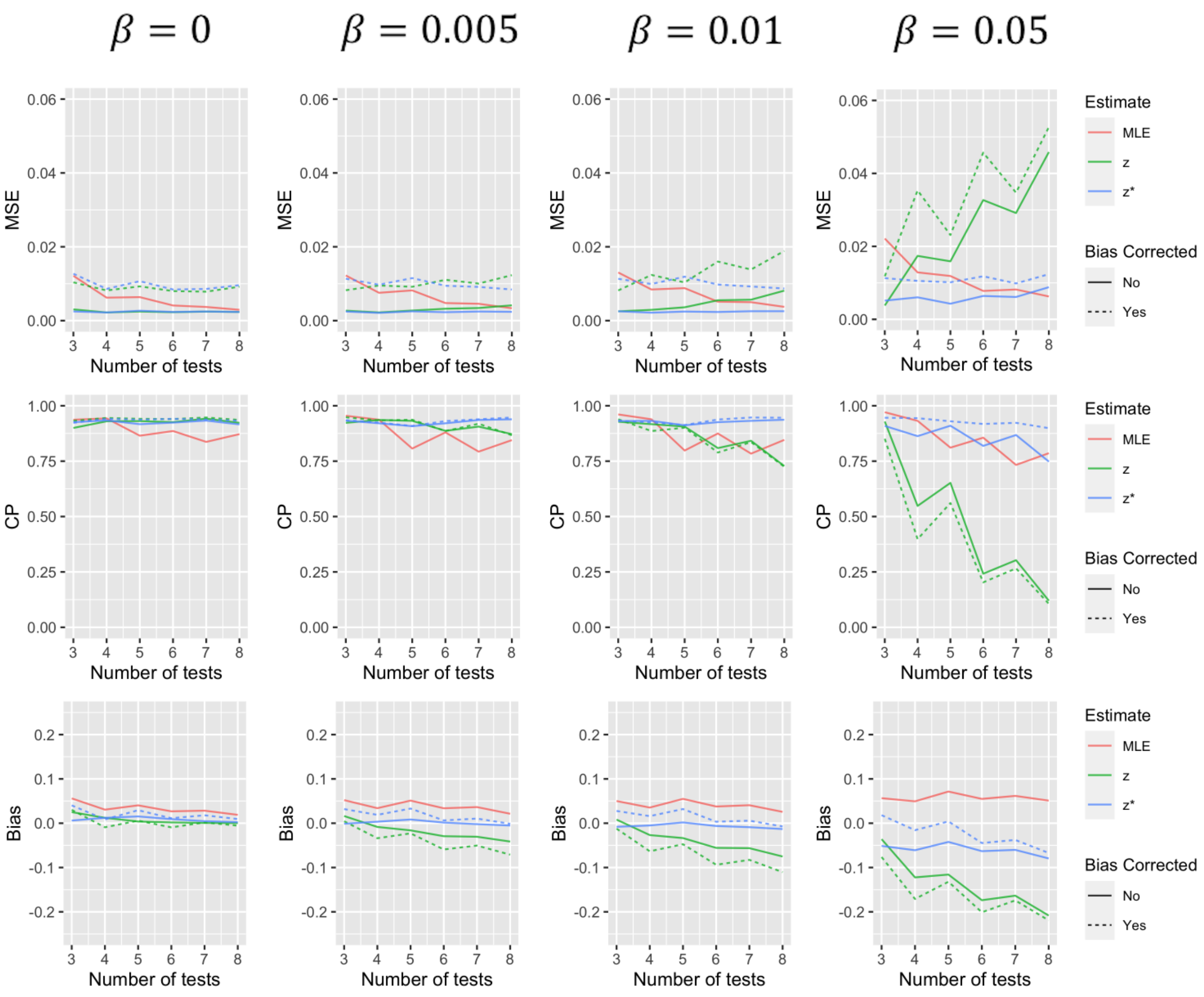

Web Figure 4: Simulation results for $\alpha(t)$ where t is the median test for scenario 2 summarizing mean squared error, coverage probability and bias of the five estimates for different values of β and varying number of tests.

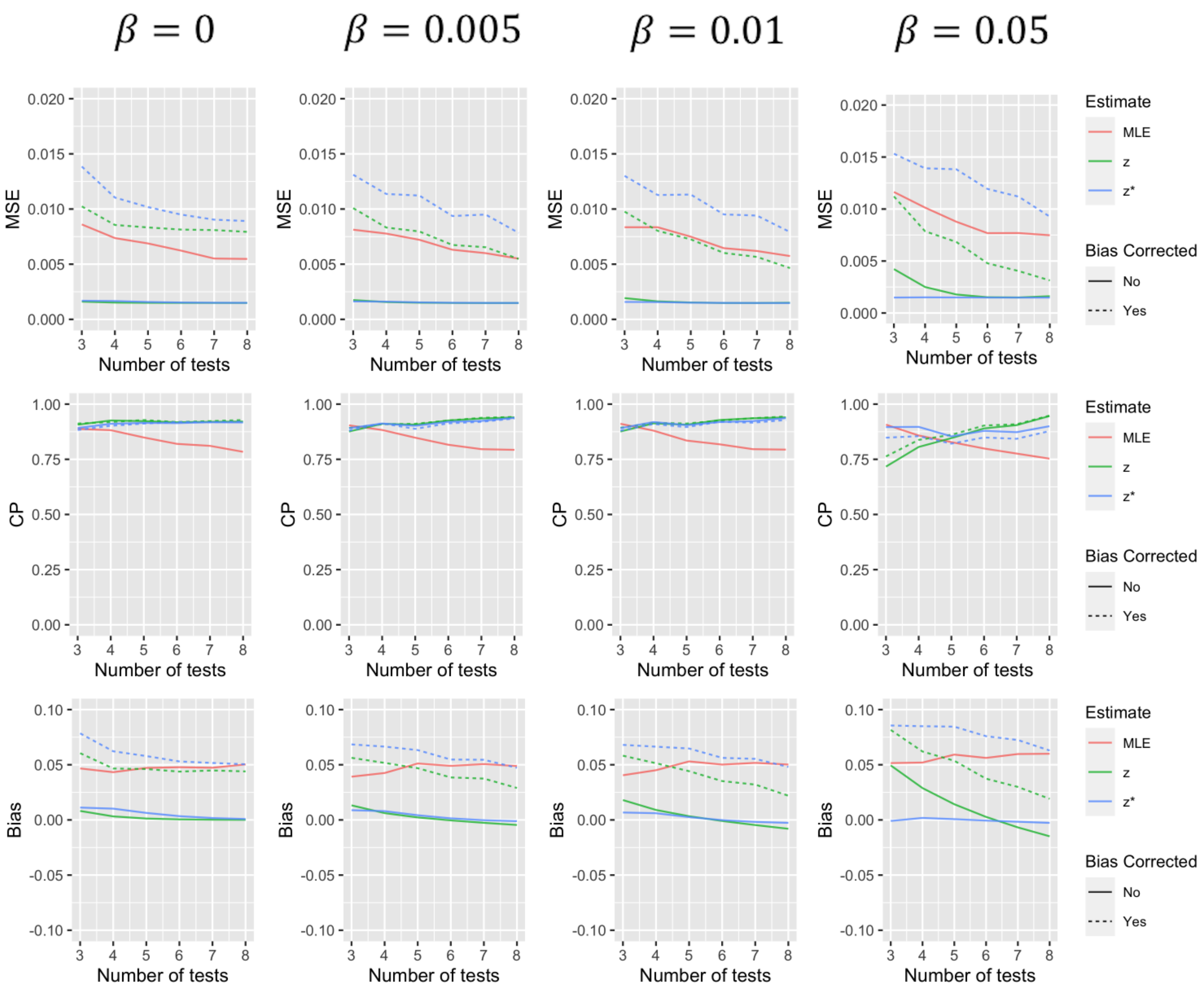

Web Figure 5: Simulation results for $\alpha(T)$ for scenario 2 summarizing mean squared error, coverage probability and bias of the five estimates for different values of β and varying number of tests.

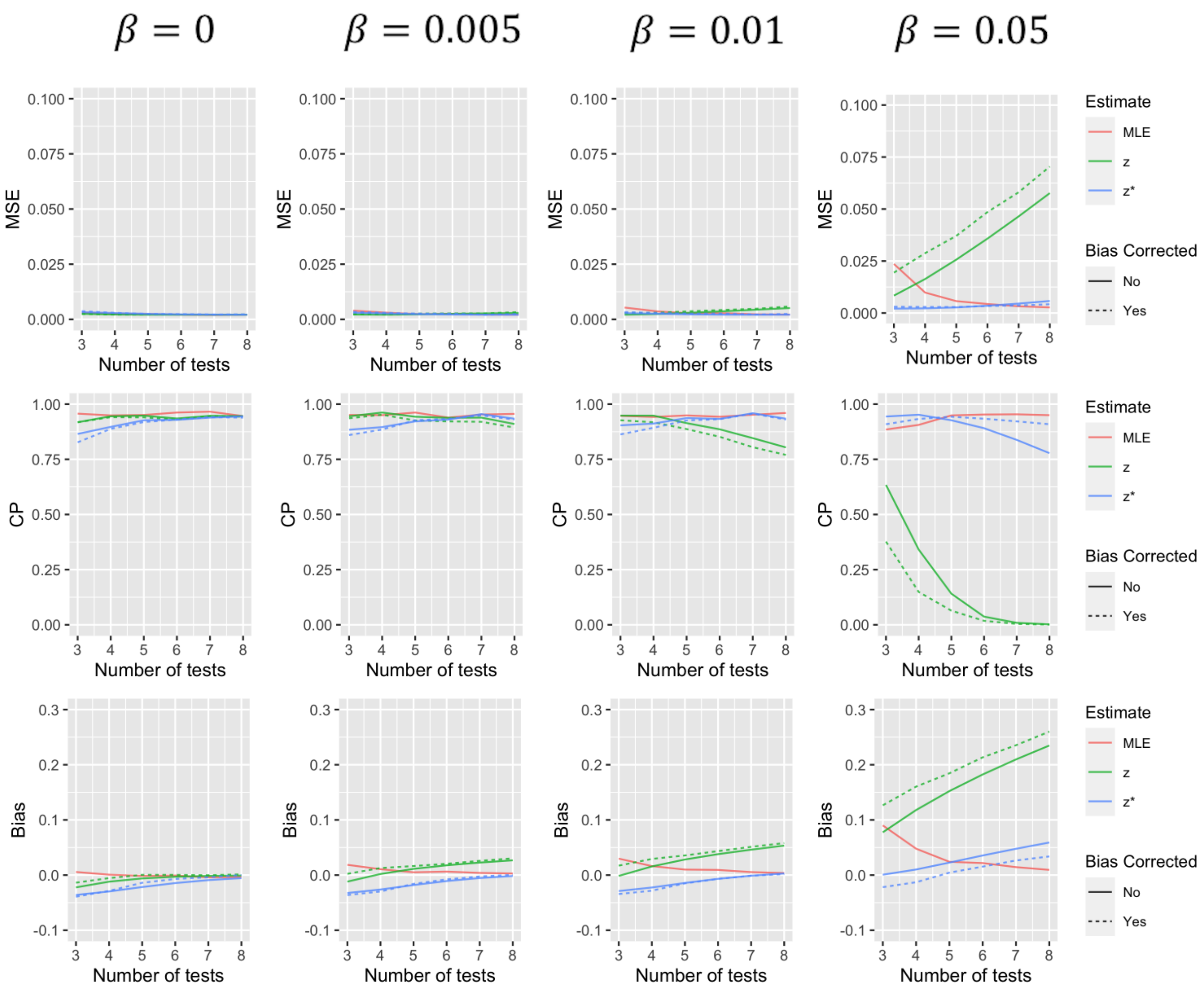

Web Figure 6: Simulation results for θ for scenario 3 summarizing mean squared error, coverage probability and bias of the five estimates for different values of β and varying number of tests.

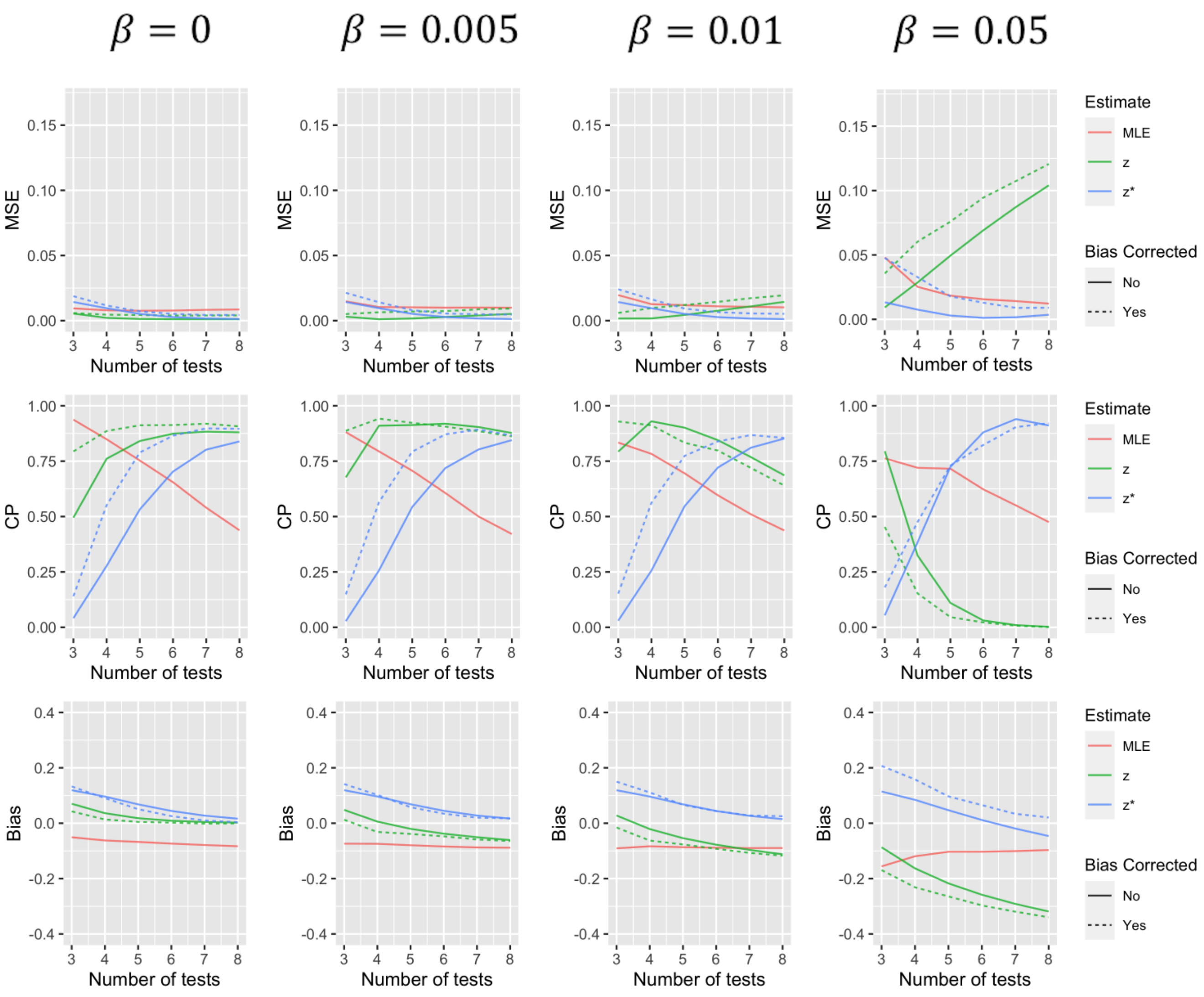

Web Figure 7: Simulation results for $\alpha(1)$ for scenario 3 summarizing mean squared error, coverage probability and bias of the five estimates for different values of β and varying number of tests.

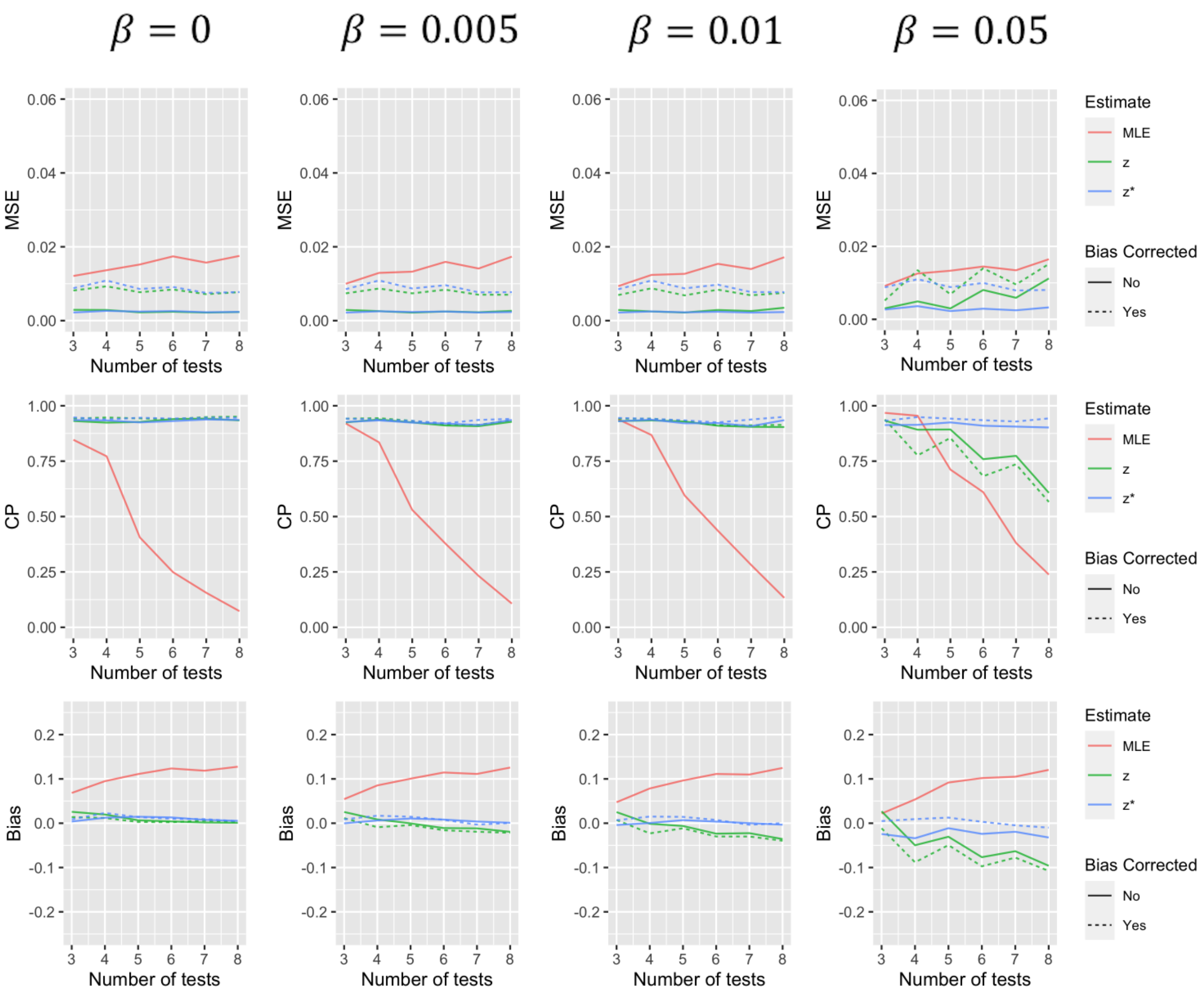

Web Figure 8: Simulation results for $\alpha(t)$ where t is the median test for scenario 3 summarizing mean squared error, coverage probability and bias of the five estimates for different values of β and varying number of tests.

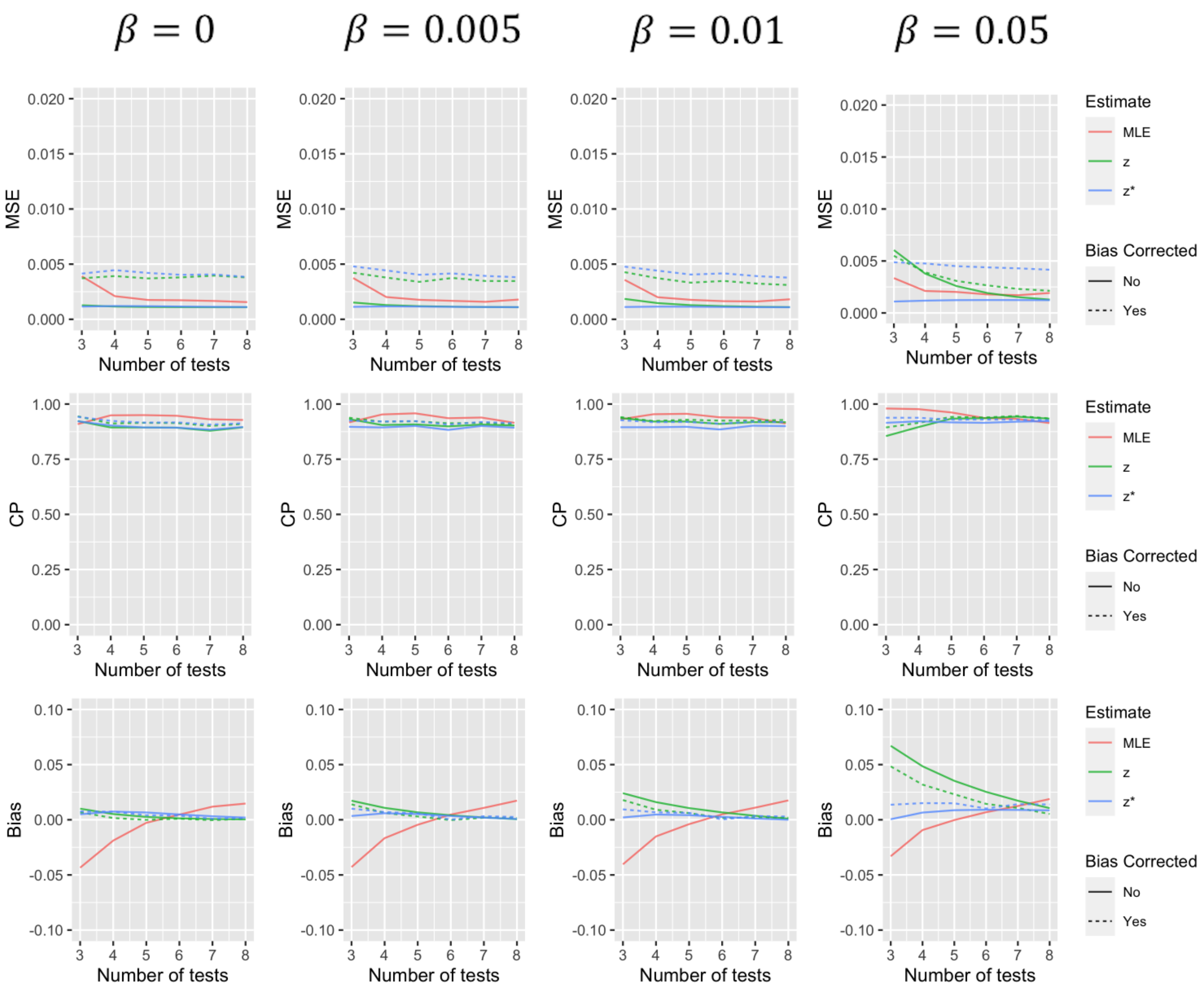

Web Figure 9: Simulation results for $\alpha(T)$ for scenario 3 summarizing mean squared error, coverage probability and bias of the five estimates for different values of β and varying number of tests.

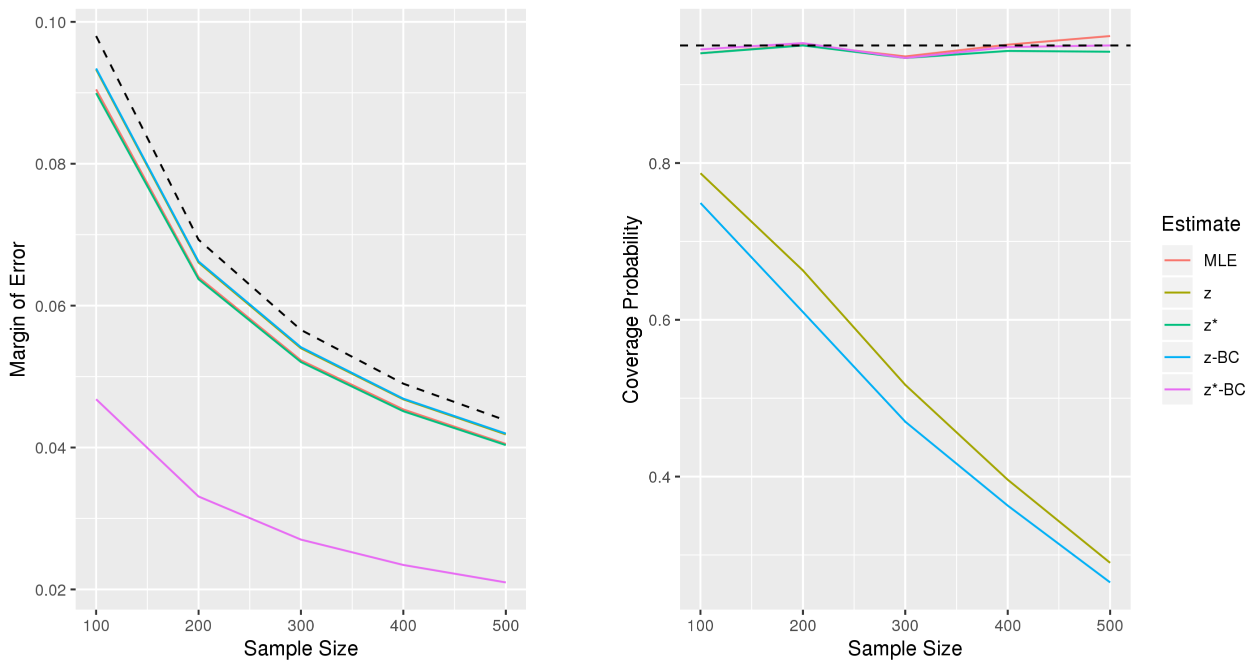

Web Figure 10: Expected margin of error and coverage probability (dashed line) and simulated margin of error and coverage probability vs. sample size for estimating θ

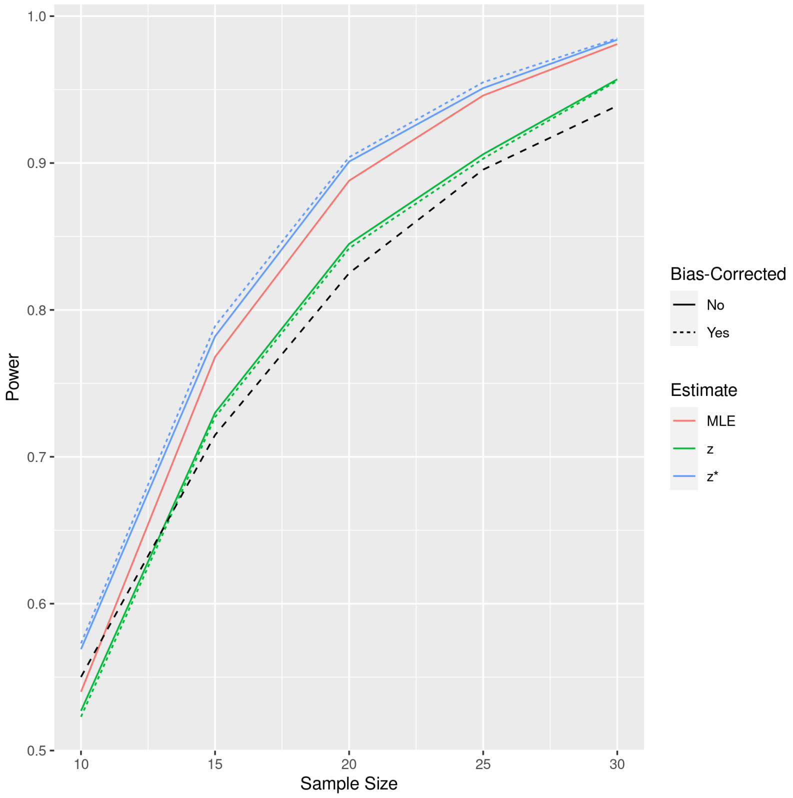

Web Figure 11: Expected power (dashed line) vs simulated power to detect decline in sensitivity over time for each estimate vs sample size.

3 Web Tables

Web Table 1: Recommended sample size to detect decline in sensitivity for given number of tests

		$T = 3$	$T = 4$	$T = 5$	$T = 6$	$T = 7$	$T = 8$
Compare coefficient to 0	80% Power	5173	1976	946	519	312	202
	85% Power	5817	2260	1082	594	358	231
	90% Power	6925	2645	1266	695	419	270
Compare DRC to Liberia	80% Power	397	153	78	48	34	27
	85% Power	455	175	90	56	39	31
	90% Power	534	206	105	65	46	36
