# Supplementary material for: Simplifying the estimation of diagnostic testing accuracy over time for high specificity tests in the absence of a gold standard
Source: Biometrics. Author manuscript; Available in PMC 2026 May 18. (PMC13181389; doi:10.1111/biom.13689)
Supplement: Drew_Biometrics_2023_zip [file NIHMS2149336-supplement-Drew_Biometrics_2023_zip.zip › Final-Code/README.html]

README


# LCM-simplification

This code accompanies the manuscript “Simplifying the estimation of diagnostic testing accuracy over time for high specificity tests in the absence of a gold standard.” The code runs a simulation to test the performance of multiple estimators for a latent class model of multiple high specificity tests with declining sensitivity over time. See the manuscript for a more detailed explanation on when these models are appropriate. The simulation specifications for the scenarios explored in the manuscript are available in a separate file and can be easily modified by the user to explore different specifications.

### Dependencies

This simulation code requires the packages dplyr, parallel, and flexmix. To recreate plots summarizing the results the packages ggplot2, gridExtra, png, and EBImage are required. All of which are all available in the CRAN library.

### Executing the program

- Download the files main\_simulation.R, simulation\_funs.R, make\_plots.R, heading.png and the parameters folder and store in your working directory
- Parameters for scenarios 1-3 with varying beta values are defined in the files within the parameters folder. E.g. s1-beta005 corresponds to the parameters for scenarios 1, with beta=0.005. These simulations can be run as is or a new parameter file can be created using these as a template.
- Note that the bootstrapping procedure for bias corrected estimates is intended to be run in parallel. Make sure to adjust the ncores parameter in the parameters file to an appropriate value for your machine (set to 1 to avoid running in parallel).
- In the main\_simulation.R file, indicate the path to the desired parameters file on line 8.
- Execute main\_simulation.R
- To create plots of simulation results similar to those in the manuscript, first adjust the scenario in the make\_plots.R file to reflect the intended scenario number (it should match the folder where simulation results are stored). Note that this file is intended to be executed after simulation results are obtained for beta = 0, 0.005, 0.01, and 0.05 but could be modified to reflect different beta values.
- Execute file make\_plots.R
