# Supplementary figures and images for: Simplifying the estimation of diagnostic testing accuracy over time for high specificity tests in the absence of a gold standard
Source: Biometrics. Author manuscript; Available in PMC 2026 May 18. (PMC13181389; doi:10.1111/biom.13689)

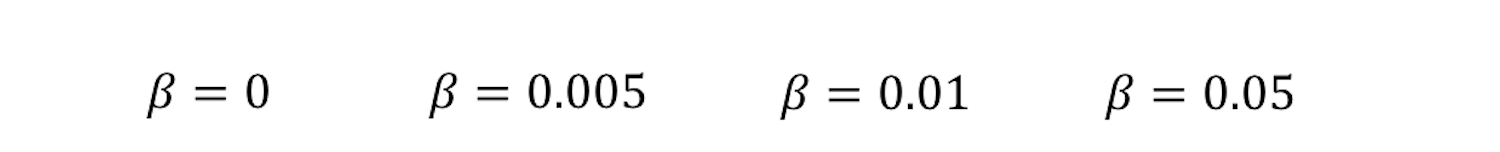

Supplement: Drew_Biometrics_2023_zip [file NIHMS2149336-supplement-Drew_Biometrics_2023_zip.zip › Final-Code/heading.png]
