# Supplementary material for: Simplifying the estimation of diagnostic testing accuracy over time for high specificity tests in the absence of a gold standard
Source: Biometrics. Author manuscript; Available in PMC 2026 May 18. (PMC13181389; doi:10.1111/biom.13689)
Supplement: Drew_Biometrics_2023_github [file NIHMS2149336-supplement-Drew_Biometrics_2023_github.docx]

The simulation R code is available both on Wiley Online Library and on Github: <https://github.com/clara-drew/LCM-simplification>.
